# Impact of Displacement on Refugee Women's Sexual and Reproductive Health: A Participatory Study Using Photovoice

**DOI:** 10.1111/1471-0528.18328

**Published:** 2025-08-19

**Authors:** Jamilah Sherally, Franceska Dnestrianschii, Zainab Alshamari, Ekram Beshir, Habibe Jafari, Masume Jafari, Kowthar Mohamed, Semira Mohammadyasin, Maria van den Muijsenbergh, Thomas van den Akker, Marielle Le Mat, Saskia Elise Duijs

**Affiliations:** ^1^ Athena Institute, VU Amsterdam Amsterdam the Netherlands; ^2^ Department of Primary and Community Care Radboud University Nijmegen the Netherlands; ^3^ Leiden University Medical Centre Leiden the Netherlands; ^4^ KIT Institute Amsterdam the Netherlands; ^5^ Amsterdam UMC, Dept. Ethics, Law and Humanities Amsterdam the Netherlands

**Keywords:** displacement, participatory research, photovoice, refugee women, sexual and reproductive health

## Abstract

**Objective:**

To explore how displacement impacts the sexual and reproductive health (SRH) of refugee women.

**Design:**

Participatory photovoice study integrating photography with qualitative inquiry.

**Setting:**

Conducted online between February and May 2024.

**Population:**

Six refugee women formerly residing in Moria Reception and Identification Centre or Mavrovouni Closed Controlled Access Centre on Lesbos, Greece, now seeking asylum across Europe and the United States.

**Methods:**

During the participatory photovoice study, participants (*N* = 6) took photographs reflecting the impact of displacement on their SRH and mental health. Visual data were explored in three focus groups and five in‐depth interviews. Participants grouped photographs into themes, with additional thematic coding by the academic researchers. An intersectional lens guided the analysis.

**Results:**

Thirty‐six photographs, many including nature symbolism, illustrated how displacement shaped SRH experiences, healthcare‐seeking behaviour and access to care for gender‐based violence, female genital mutilation/cutting, family planning and menstrual, maternal, and gynaecological health. Eight themes emerged: bodily autonomy, instability, living conditions, social support, celebrations, healthcare access, resilience, and finding purpose. While displacement exacerbated SRH needs and undermined bodily autonomy, the instability of the asylum process led to deprioritisation of healthcare‐seeking. Illness was only experienced until a sense of safety was established. Access to healthcare was compromised by language barriers, undignified treatment, and financial constraints. Despite challenges, narratives highlighted resilience, community, and personal growth.

**Conclusions:**

Trauma‐informed, culturally sensitive healthcare is essential for SRH equity among refugee women. Refugee‐led visual research offers a transformative tool for knowledge production and advocacy.

## 1 Introduction

Refugee and asylum‐seeking women (henceforth referred to as refugee women) face disproportionately poor sexual and reproductive health (SRH) outcomes compared to host populations, including higher rates of pregnancy complications [1, 2], maternal mortality [3], sexually transmitted infections (STIs) [3, 4], and exposure to gender‐based violence (GBV) [5, 6]. Throughout displacement, access to SRH services is often fragmented, de‐prioritised, or unavailable [7, 8, 9]. These inequities are shaped by a complex interplay of legal precarity, language barriers, economic hardship, systemic discrimination, stigma, and restrictive gender, social and legal norms that govern how and when women can seek care [10, 11, 12, 13, 14]. Health is often deprioritised during migration, while trust in services remains low due to limited health literacy and the absence of culturally appropriate, gender‐sensitive care [7].

The absence of robust participatory, refugee‐led, data to guide SRH responses may explain these persistent disparities [8, 15]. Participatory action research aims to include those whose lives are subject to the study in all phases of the research process [16]. Unfortunately, refugee women remain underrepresented in research and largely excluded from shaping the agendas that define their health [17, 18, 19]. Furthermore, how refugee women are perceived—by policymakers, healthcare providers, and wider society—affects their access to services, autonomy in decision‐making, and ability to advocate for their needs [20, 21]. Thus, in order to improve SRH outcomes for refugee women, there is an urgent need for research that centres their perspectives.

Forced displacement is not a singular event but an ongoing process, influencing health trajectories long after initial migration [20]. Understanding how displacement shapes refugee women's SRH is crucial for developing healthcare systems that are responsive to lived realities beyond refugee camps. While displacement is recognised as a determinant of health [20], few studies have explicitly explored its impact on SRH trajectories over time [22], and to our knowledge, none have employed participatory visual methods. Photovoice, as such a participatory method, serves three key purposes [23]. First, it enables participants to express and engage with their stories on their own terms. Second, it creates a dialogical space where individual experiences are collectively discussed within the context of broader structural inequities. Third, photovoice fosters communal engagement, allowing participants to challenge experiences of marginalisation through advocacy efforts.

This study addresses the above knowledge and methodological gaps and responds to growing calls for both the meaningful participation of refugees [17], and the application of a gender‐justice (The Lancet Commission on gender and global health defines gender justice as ‘encompassing the realisation of universal rights in relation to health equity and gender equality, while also addressing the drivers of gender‐based discrimination and exclusion’) lens [24] in global health research. Using photovoice, we explore the impact of displacement on refugee women's SRH, examining how multiple social identities interact with structural and contextual determinants of health. The study is grounded in feminist principles (seeking to understand women's lived experiences in their own words and allowing for knowledge production beyond existing hierarchical structures [25]), employs intersectionality as an analytical lens (recognising how intersecting power hierarchies and identities like race, economic status and gender create both privilege and oppression [26]) and participatory action research as its methodological foundation. By centring refugee women's lived experiences as both the subject and lens of analysis, the findings aim to inform policy, support service provision and promote both social and epistemic justice.

## 2 Methods

### 2.1 Study Design

By integrating visual storytelling with qualitative inquiry, this photovoice study explored the SRH needs of refugee women over time and across different settings. Photovoice is an arts‐based approach which focuses on people's lived experience (phenomenology) in combination with a collective reflection on how these experiences are structurally embedded, using critical theories such as intersectionality [26]. Findings from the current analysis are part of a broader participatory mixed‐methods study that aims to provide a comprehensive understanding of refugee women's SRH status, needs, and access to healthcare in Closed Controlled Access Centre (CCAC) Mavrovouni on Lesbos, Greece.

### 2.2 Patient and Public Involvement

Six refugee women with lived experience in Moria Reception and Identification Centre (RIC, Europe's largest refugee camp, destroyed by fires in September 2020) and/or Mavrovouni CCAC (which replaced RIC Moria) on Lesbos participated in the entire process: from developing the research question to analysing and disseminating the results. They are also co‐authors on this paper.

To co‐create the research question, participants were invited to identify themes they wished to explore further. In addition to the impact of displacement on SRH, they highlighted mental health and transience. These themes informed the prompt for photographs, namely: ‘What is the impact of being on the move and the asylum‐seeking process on your mental and sexual and reproductive health?’

### 2.3 Setting

The study was conducted online via Zoom between February and May 2024, enabling participation across different countries. Participants took pictures in their own environments (Table 1). A pre‐existing WhatsApp group was used for initial contact, communication, and study updates.

**TABLE 1:** Participant demographics.

Participant	Age	Origin country	Location as of May 2024
Participant 1	26	Eritrea	USA
Participant 2	27	Ethiopia	Switzerland
Participant 3	26	Somalia	Germany
Participant 4	22	Iraq	Germany
Participant 5	28	Afghanistan	Germany
Participant 6	30	Afghanistan	Germany

### 2.4 Participants

Participants of this study were identified during an online reunion of nine refugees previously involved in a mixed‐methods study on Lesbos. These nine co‐researchers were originally recruited through Non‐Governmental Organisation (NGO) and refugee networks, social media channels, and word‐of‐mouth. Inclusion criteria included identifying as a woman, being (digitally) literate and proficient in English, having lived experience in Moria RIC and/or Mavrovouni CCAC, and being available and motivated to commit to a co‐researcher role. At the time, co‐researchers were purposefully selected to represent the main ethnic groups residing in CCAC Mavrovouni (Afghan, Eritrean, Ethiopian, Somali, Iraqi, and Congolese), following interviews conducted by the principal investigator J.S. During the research period on Lesbos, the idea of visual documentation emerged organically, as co‐researchers expressed frustrations about the limitations of conventional research in capturing their lived experiences. Since the completion of data collection in September 2023, team members had relocated to countries outside Greece but remained in touch via WhatsApp.

During the online reunion in February 2024, J.S. invited the co‐researchers to expand their previous work through the visual storytelling they had previously proposed. F.D. and S.E.D. introduced photovoice as a method. Six women (Table 1) agreed to participate, and verbal consent was obtained and recorded. In contrast to the study on Lesbos, no financial compensation was offered, mutually agreed upon as acceptable.

### 2.5 Data Collection

Previously on Lesbos, the six participants had received a three‐week training covering research methodologies, ethical considerations (informed consent, confidentiality) and trauma‐informed interviewing. They also had prior experience facilitating community focus group discussions (FGDs) that employed photovoice to explore barriers and facilitators to SRH care in CCAC Mavrovouni. For this study, they received further training by S.E.D., learning how to incorporate colour, contrast, symbols, and shapes to enhance photographic expression.

Participants took photographs on their mobile phones, titled them to reflect their intended message, and shared these in the communal WhatsApp group. J.S. and F.D., supported by S.E.D., facilitated discussions on the photographs during three FGDs (the first and last authors are trained photovoice facilitators). As the project progressed, it became evident that smaller group settings encouraged deeper engagement, with participants sharing more detailed narratives. In response, individual in‐depth interviews (IDIs, *N* = 5) were conducted by F.D. to allow for a richer exploration of their photographs.

FGDs were conducted online with cameras enabled when internet connectivity allowed, and participants joined from private spaces using their personal devices. All discussions were held in English, the common language among the team, and were audio‐recorded with permission. Transcriptions were completed verbatim. In line with our photovoice approach, we critically reflected on lived experiences and how these were shaped by social determinants of health and intersecting identities [27, 28, 29]. FGDs lasted approximately 1.5 h, while IDIs averaged 1 h each.

### 2.6 Data Analysis

During the final FGD, participants categorised photographs based on commonalities within the accompanying narratives. They led the analysis with F.D. sorting the images virtually on a shared screen. When there were disagreements, follow‐up questions were asked until the group reached consensus. Some photographs were sorted into multiple categories.

The final selection of photographs for exhibition was collectively determined: each participant voted on 15 photographs which they felt were most important to show to a wider audience, with J.S. and F.D. ensuring that all key themes were represented and every participant's perspective included. Only three participants consented to having their names displayed alongside their photographs, leading to a joint decision to omit names.

Alongside participant‐led analysis, J.S. and F.D. independently coded the transcripts using AtlasTi. A codebook was developed inductively, cross‐checked with participant‐generated themes, and refined through discussions with S.E.D. Analytical disagreements were presented to S.E.D., M.L.M., M.M., and T.A. and resolved during team meetings. Intersectionality was applied as a critical lens, examining how identity dimensions and systemic marginalisation shaped participants' experiences [26, 30]. The final analysis triangulated the coded transcripts, photographs, and participant‐generated themes.

### 2.7 Ethical Considerations

Ethical approval was granted by the Hellenic National Public Health Organisation under reference number 25298/23‐12‐2022. Throughout the study, emphasis was placed on voluntary participation, with guarantees of confidentiality, secure data storage, and the right to withdraw photographs without consequence. In addition to verbal consent for participation, all participants gave explicit, recorded verbal consent for the public use and publication of their selected photographs, including exhibition and academic dissemination. Transcripts and photographs were anonymised, ensuring that participants and external individuals were unidentifiable.

## 3 Results

Thirty‐six photographs were collected (Figure S1). Two photographs were excluded following group consensus: one for safety concerns, one due to lack of explicit consent. Titles were chosen by participants, accompanying texts by J.S. While exhibitions were part of the photovoice project, they fall outside the scope of this paper and are therefore not presented here.

Photographs primarily depicted narratives on mental health, GBV, including sexual violence, child marriage, and female genital mutilation/cutting (FGM/C), family planning and menstrual, maternal, and gynaecological health. Participants categorised photographs into eight themes: bodily autonomy, instability, living conditions, social support, celebrations, healthcare access, resilience, and finding purpose. In the following sections, we, the academic researchers, present findings in four overarching categories illustrating how displacement impacts refugee women's SRH experiences, needs, health seeking behaviour, and healthcare access. Table S1 offers an overview of the qualitative analysis with accompanying photographs and exemplary citations.

### 3.1 Intersecting Identities Shaping Sexual and Reproductive Healthcare Experiences

Testimonies revealed how SRH experiences were shaped by intersecting identities, including gender, race, culture, religion, disability, socioeconomic status, power dynamics, and refugee status. One participant's experience on a train vividly illustrated how these overlapping factors heightened health inequities.

**FIGURE 1:**
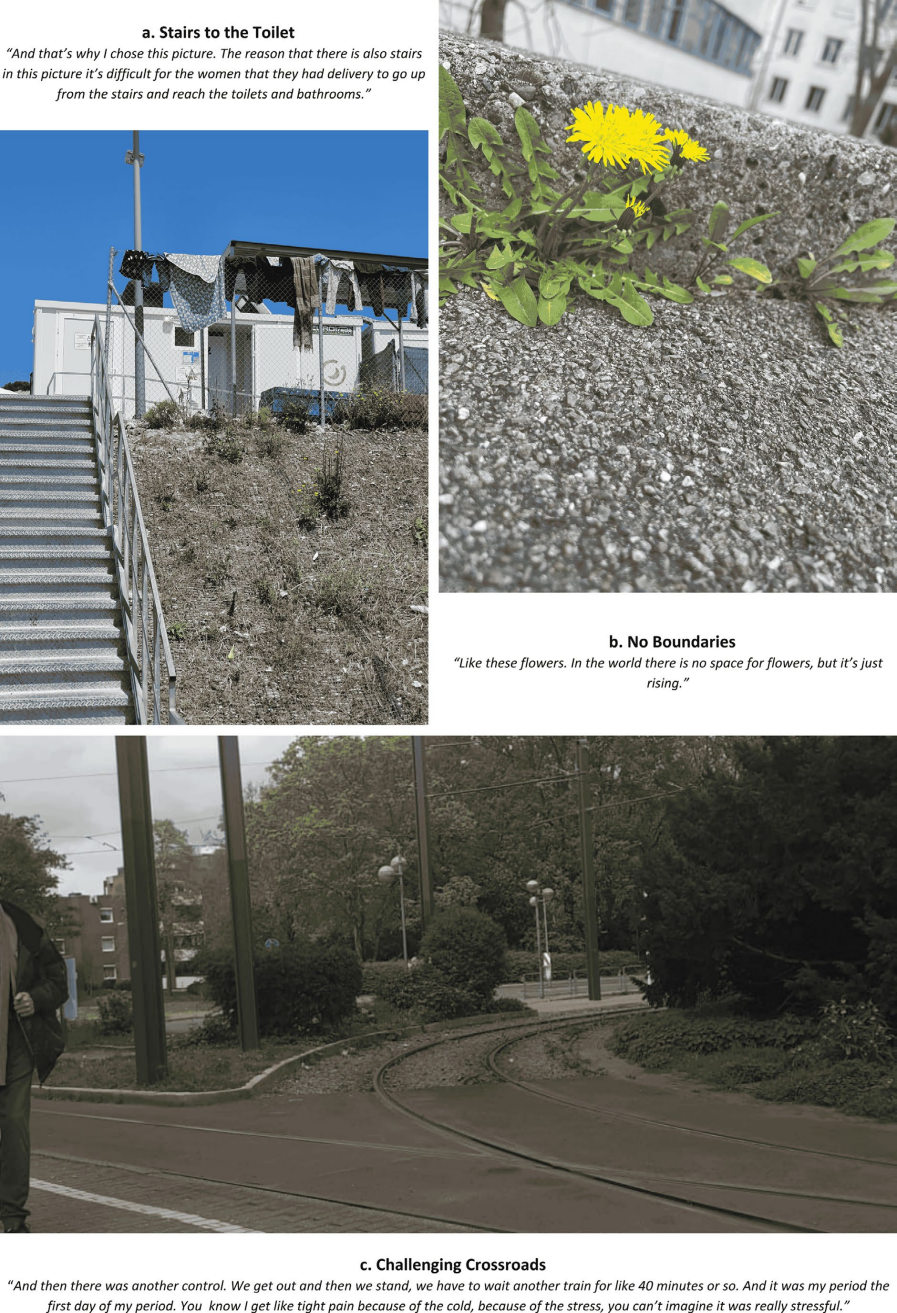
Collage of photographs depicting SRH experiences.

She described severe menstrual pain while travelling, exacerbated by FGM/C and the inability to afford a ticket, leaving her exposed to the cold for extended periods. When she eventually boarded, the conductor shouted at her, further intensifying her distress. Her photograph (Figure 1c) was thus a reflection of multiple health disparities converging to shape her menstrual health experience: gendered harm (FGM/C‐related pain), economic precarity (inability to afford transport), and power imbalances (humiliating treatment by an authority figure) possibly compounded by race (she was a black woman). Her account of seeking employment was similarly layered:

You know for me I have FGM and when I get my period it's super pain! Sometimes I start crying, crying until I get sleep. [That day I was looking for a job] They just written down your name and nobody talk to me, I just standing there for around one hour and nobody talks to me. Everybody works inside the company and I'm outside.

Disability and refugee status also shaped SRH experiences, particularly within the constraints of shared accommodation. A photograph of stairs (Figure 1a) illustrated the difficulty of accessing toilets after caesarean‐section. Others captured the impact of extreme cold (30) on menstrual regularity, or shared bathrooms on menstrual hygiene (3). Shared living spaces also compromised mental health, exemplified by symbols of rain and mountains which offered repose for participants to leave the asylum centre and ‘go there and shout and then think for a while.’

At the same time, participants reflected on resilience in relation to mental health, particularly through religious identity. Various photographs (7, 24, 35) referenced nature as a parallel to life's hardships—seasons changing, landscapes transforming—reinforcing the belief that suffering was temporary (Figure 1b). As one participant explained:

You know, Allah says this life is hard for the moment. So, it's hard for us, and I accept it. […] Yeah, it's normal. Just for two years, and a few years later, it's temporary.

### 3.2 Navigating Healthcare Access

**FIGURE 2:**
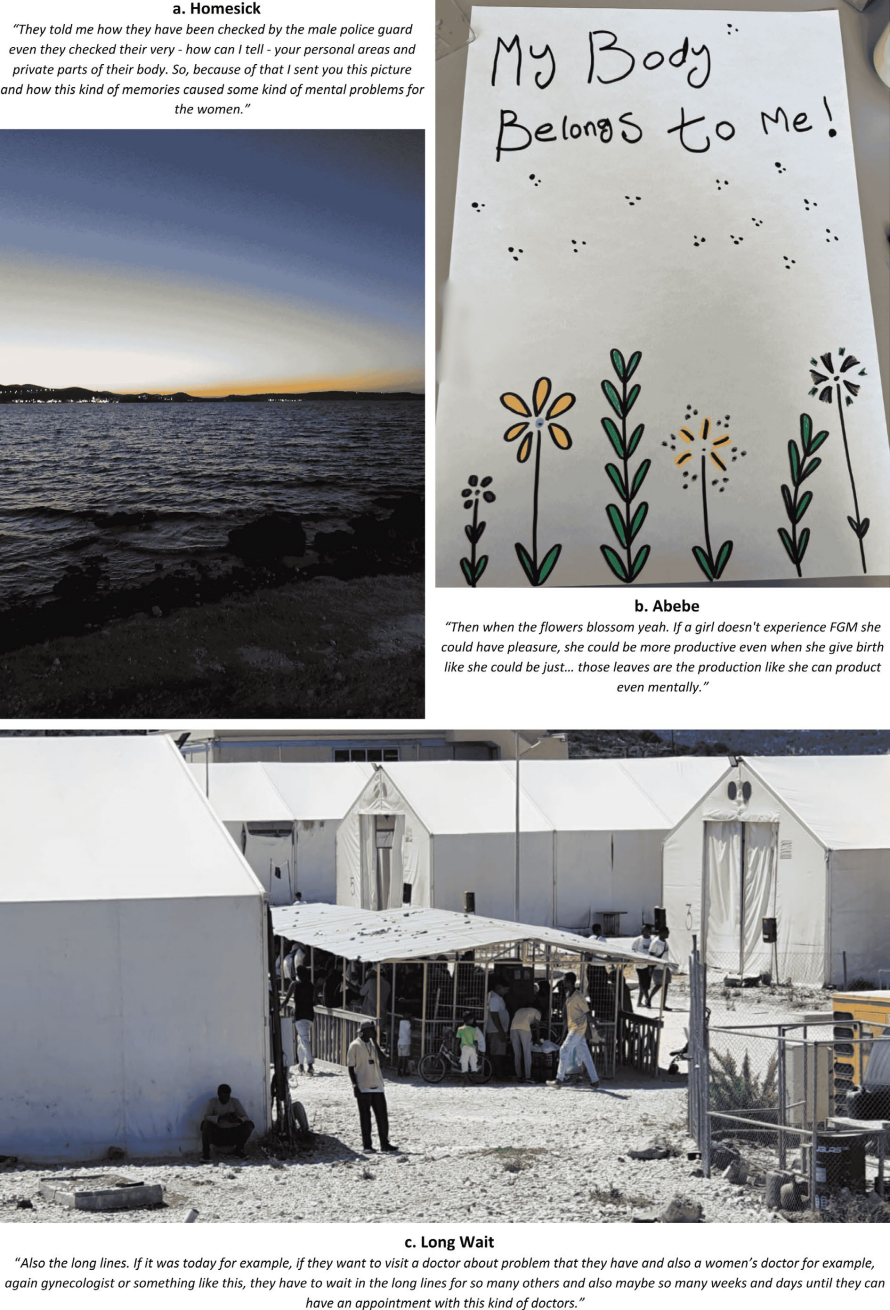
Collage of photographs depicting healthcare access.

Healthcare access was characterised by participants' hope of attaining bodily autonomy in resettlement countries, yet confrontation with access barriers. Displacement undermined both bodily autonomy and mental health through coerced early marriages (often undertaken as economic survival or a strategy to secure asylum (2)) and sexual violence during travel (11, 20), which participants described using ocean metaphors (Figure 2a). Several photographs, often featuring flowers, focused on FGM/C and the need for care. For instance, ‘Hope’ (2) depicted an information booklet educating women about their rights. The participant elaborated on her title:

There's a lot of struggling with the FGM. You know some people they complaining that they lost all of their clitoris or every part of their vagina. […] But now here there is a surgery like free and the government support them if they need to make a reconstruction.

Figure 2b of blossoming flowers conveyed both resistance to FGM/C*—*‘I want to tell the parents not to do that. Like those flowers: if we keep them, our flowers could be beautiful…’—and the potential for improved mental and SRH if effective healthcare, such as free reconstructive surgery, became accessible.

Then when the flowers blossom yeah. If a girl doesn't experience FGM she could have pleasure, she could be more productive even when she give birth. Like those leaves are the production, like she can product even mentally.

Despite these images of hope and potential, participants highlighted persistent barriers. Fenced areas (12, 22, 34) and concrete structures (13, 24) symbolised not only physical confinement in asylum centres but also obstacles to access: language barriers (1, 25, 32), waiting lines, financial constraints (9, 16, 17), undignified treatment (15, 33), and dismissive attitudes by health professionals (33).

### 3.3 The Impact of Displacement on Healthcare Seeking Behaviour

Photographic narratives revealed how the instability of the asylum process disrupted mental health and influenced healthcare‐seeking behaviour. Participants likened displacement to an ‘earthquake’ (7) and spoke about the duality of the ocean (Figure 2a)—a source of calm yet also a reminder of the deadly risks of sea crossings and the trauma of GBV during travel.

In my mind it's like this: the sunsets and the sky is beautiful but in the contrast the sea can die the people. Even though the sea can make you feel calm and can give the people peace, but on the other hand it can be very dangerous and be like a murderer

**FIGURE 3:**
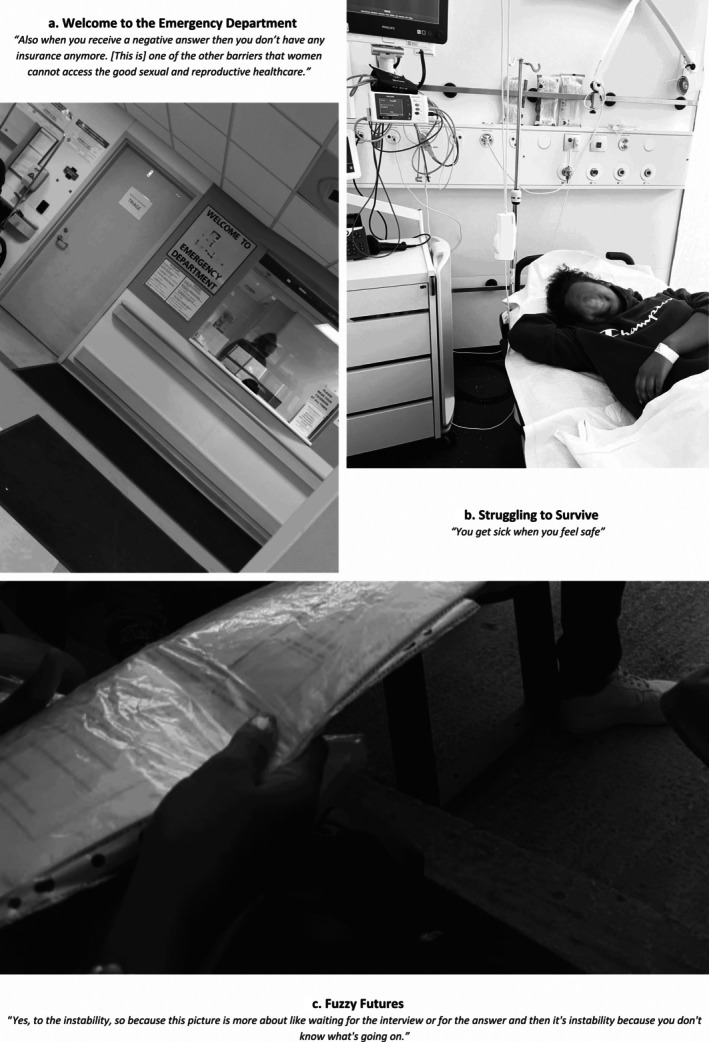
Collage of photographs depicting healthcare seeking behaviour.

Instability not only eroded mental wellbeing but also shifted priorities from seeking timely care. Participants described how uncertainty over their legal and financial status caused them to deprioritise healthcare (Figure 3c), only seeking care in emergency situations (Figure 3a). One reflected on her gynaecological symptoms: ‘Yes, I have to give the first priority for my health. But in this moment I can't, because the first thing is I have to give the priority for my work.’

Perceived safety influenced healthcare seeking behaviour. Participants shared that they only allowed themselves to fall ill once they felt secure (Figure 3b). ‘You get sick when you feel safe,’ one noted, describing how illness emerged after reaching stable environments. Another elaborated, ‘She had to be strong and don't get sick until she can be in a safe place. It happens to most of the people when they arrive after some years of being on the move’.

### 3.4 The Importance of Recognition and Informal Networks in Foreign Formal Systems

**FIGURE 4:**
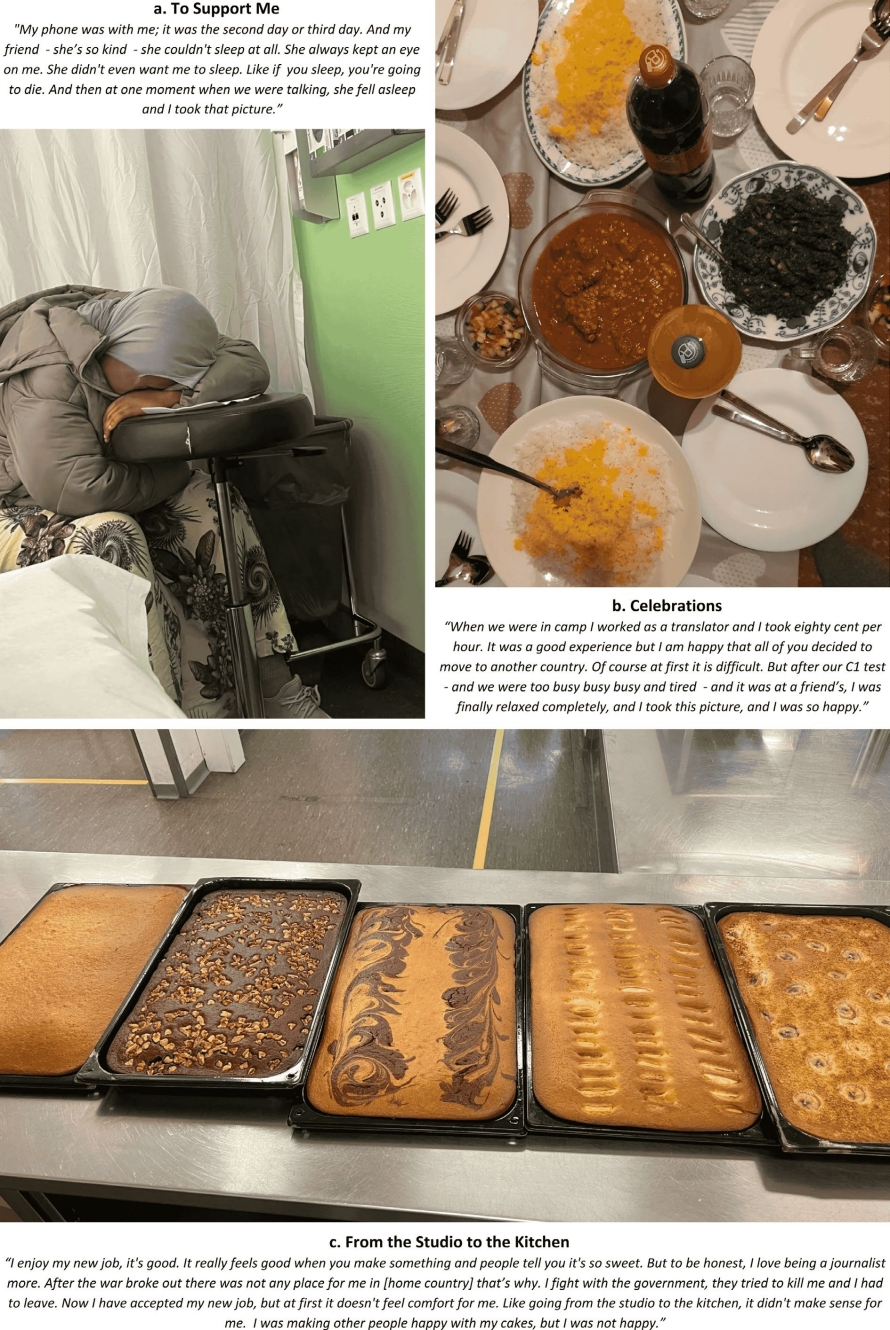
Collage of photographs depicting social determinants of SRH.

Refugee women's healthcare needs extended beyond formal clinical services, encompassing social support and recognition. Informal care networks, often gendered in nature, played a key role in both social and health‐related support. Participants not only mentioned the importance of their current social networks but also spoke with nostalgia about past relationships using imagery of fenced sunsets (20, 22) and fenced groups of flowers (35). One participant shared how a friend remained by her side during hospitalisation, too afraid to sleep in case something happened (Figure 4a):

She's so kind—she couldn't sleep. She always kept an eye on me. She didn't even want me to sleep. Like if you sleep, you're going to die.

In relation to mental wellbeing, participants described how recognition (1, 4, 28, 33), work (4, 5, 33), and skill development (25, 27, 29, 32) were essential. Celebratory images—such as Figure 4b, depicting a language acquisition milestone—symbolised progress toward self‐sufficiency and integration into new communities (25–27, 31). The importance of validation extended toward formal SRH services. Especially for higher‐educated participants, moments of professional recognition helped counter feelings of exclusion. One participant, a public health graduate, described the significance of being acknowledged for her ability to interpret an ultrasound image of a foetus (1). Two other participants explained how work contributed to self‐worth, but expressed frustration at working below their capacities as a journalist (Figure 4c) and computer specialist (5). Reflecting on the broader gendered, classed, and racialised inequities, one remarked:

I think they think that refugees don't study… they don't know anything. And I really felt frustrated and really disrespected, you know?

## 4 Discussion

### 4.1 Main Findings

Our study highlights how displacement shapes refugee women's SRH experiences, healthcare‐seeking behaviour, and access to care. Photographic narratives underscored how SRH needs included broader determinants, such as informal social networks, to navigate foreign formal systems. While displacement exacerbated GBV needs in particular, the instability of the asylum process resulted in deprioritisation of health, and healthcare‐seeking was often delayed until a sense of safety was established. Hope for bodily autonomy and FGM/C treatment in resettlement countries was prominent. However, access was often hindered by language barriers, undignified treatment, and financial constraints. SRH experiences were shaped by intersecting factors such as gender, culture, and socioeconomic status. Despite challenges, photographs also documented resilience, with participants finding purpose and celebrating personal growth in new environments.

### 4.2 Strengths and Limitations

While participatory research with refugees is steadily gaining traction [19], and photovoice has been deployed in various refugee contexts, their application within the field of SRH remains limited. Only a handful of studies have employed participatory approaches to explore refugee SRH [31, 32], and to our knowledge, just one utilised photovoice (to explore pregnancy care among Karen women of refugee background in Australia) [33]. This study is therefore distinct in scope, approach and context. A key strength of this study was the pre‐existing rapport among the research team, which enabled immediate engagement with the subject matter. The safe space established through prior collaboration allowed for open, unguarded discussions. Beyond data collection, sessions fostered connection: participants exchanged personal struggles, celebrated milestones, laughed and cried together. Consequently, and aligned with the intent of participatory research, the sessions served as both an intervention and a research method [28]. Moreover, it reinforced our finding regarding the role of social support as a determinant of SRH.

Our methodology allowed for the collection of thick qualitative data [34], as participants narrated their experiences with minimal prompting. The absence of rigid guidance on photography or predefined themes ensured an unobstructed, participant‐led exploration of all facets of SRH. The results were photographs that depicted both the complexity and nuance of participants' lives, challenging the prevailing, often homogenised, depictions of refugees as either victims or heroes [35], and underscoring the value of an intersectional lens in understanding their experiences [30]. A key strength of photovoice is that it allows people to capture both their communities' needs as well as assets [23]. As such, celebrations emerged as a prominent theme in our study, as did social networks and the desire for recognition beyond refugee status.

The virtual format, while enabling geographically dispersed participation, introduced technical challenges, including WiFi disruptions and difficulty viewing materials on mobile devices. Fluctuating attendance occasionally resulted in smaller FGDs. However, this limitation proved beneficial, enabling deeper reflection and prompting a transition to IDIs. Similarly, frequent interruptions offered valuable insights into the realities of limited privacy in asylum centres.

The use of convenience sampling meant participants were all digitally adept and well‐educated, possibly limiting the generalisability of findings. The sample was also relatively young and consisted mainly of individuals in the post‐camp phase of the asylum process, which may have shaped the perspectives shared. A more diverse age range, including women with children and care‐giving responsibilities, might have yielded additional insights. Nonetheless, participants demonstrated reflexivity, frequently contrasting their experiences with those of peers who faced greater barriers related to finances, language, digital skills, and social support. Furthermore—drawing from their prior research experience on Lesbos—they often contextualised their personal narratives within broader refugee experiences, exemplifying the ‘critical consciousness’ that photovoice seeks to foster [36].

### 4.3 Interpretation

Our findings align with existing literature on the mental health impact of the asylum process [37, 38, 39] the importance of social support [40], and the role of resilience among refugee women [41, 42, 43]. While our earlier review on SRH access for refugees transiting through Europe also concluded that many prioritise travel over immediate health needs [7], this study offers a more nuanced explanation: women may only fall ill and seek healthcare when a threshold of perceived safety is reached. In European contexts, asylum seekers' high use of emergency care is often attributed to limited health system literacy, language barriers, or other structural obstacles [44, 45, 46, 47, 48]. Our findings reframe delayed care as a complex coping strategy shaped by intersecting factors of class (financial constraints), gender (re‐traumatisation of GBV during police checks), and legal status (asylum insecurity). Clinically, this insight suggests that if illness is tied to perceived safety, healthcare providers should adopt trauma‐informed approaches that build trust and emotional security. From a policy perspective, it challenges the narrow focus on structural barriers and calls for holistic interventions that integrate psychosocial support with medical care.

Although the importance of trauma‐informed SRH care (TIC‐SRH) has been recognised [49], its implementation and scale‐up remain complex. There is a lack of clear guidance on what constitutes high‐quality TIC‐SRH in resettlement countries [50], underscoring the need for a comprehensive, context‐specific framework [49]. Embedding TIC often demands structural reconfiguration; retrofitting it into systems that perpetuate the language barriers, long waiting times, financial constraints, and undignified or dismissive care identified in our study risks undermining its core principles of agency, dignity, and trust. However, our participatory approach demonstrates that involving refugee women from the outset can help embed TIC principles in meaningful ways. Realising this at scale will require sustained investment, staff training, and political commitment to redesign healthcare systems around the lived realities of displacement.

Without photovoice the significance of nature symbolism might have gone unnoticed. While previous studies have described embodied narratives of loss and trauma among refugees [51, 52, 53], the role of natural elements (e.g., flowers, sunsets, the ocean, earthquakes, mountains, rain) in articulating SRH needs appears absent from the existing literature. Recommendations regarding intercultural SRH communication in resettlement contexts often emphasise translation services and practitioner's cultural competence [49, 54, 55], with little attention to the role of symbolism. Our findings suggest that clinicians should be attentive to visual and metaphorical expressions when engaging with refugee women's SRH.

Similarly, the finding that participants actively desired bodily autonomy and FGM/C care is significant, as it highlights the often‐overlooked dimension of agency. Despite substantial research on the attitudes, consequences and clinical management of FGM/C [56, 57, 58, 59, 60, 61], women's actual needs and autonomous efforts to obtain specialised care have hardly received attention. The shift from viewing refugee women solely as victims of FGM/C to recognising them as active agents underscores the need to move beyond cultural analyses and focus on their lived realities. Clinically, this insight calls for healthcare systems that proactively listen to refugee women's needs and highlights the importance of culturally sensitive, trauma‐informed FGM/C pathways.

## 5 Conclusion

Displacement shapes refugee women's SRH experiences, needs, healthcare‐seeking behaviour, and access to care through intersecting factors. Understanding these complex realities requires shifting from generalised accounts to situated, lived experiences. SRH responses should integrate clinical care with interventions that promote social support, economic opportunity, and personal development. Such approaches not only address immediate health needs but also foster resilience and mental wellbeing—essential in contexts of chronic instability. Recognising that refugee women may delay care until they feel safe could help clinicians adopt trauma‐informed approaches that account for the psychosocial context of displacement. Particularly in relation to GBV, clinicians should be attuned to metaphorical expressions which may carry meaning for refugee women. Equally important is the recognition of refugee women beyond their legal status. Meaningful engagement that acknowledges their agency is crucial in fostering dignity and inclusion within healthcare settings. Further research can inform the development of guidelines and training that promote such empowering care practices.

## Author Contributions

J.S. was the Principal Investigator and conceptualised the study. Z.A., E.B., H.J., M.J., K.M. and S.M. developed the research question, collected the data, and conducted a first analysis of results. J.S., F.D. and S.E.D. coordinated data collection and management. J.S. and F.D. completed an in‐depth analysis of the data, with input from S.E.D., M.L.M., M.M. and T.A. J.S. wrote the first draft of the paper with input from all other authors.

## Ethics Statement

Ethical approval was obtained from the Hellenic National Public Health Organisation of Greece (reference: 25298/23‐12‐2022).

## Conflicts of Interest

The authors declare no conflicts of interest.

## Supporting information


**Figure S1:** Collection of photographs.

**Table S1:** Qualitative analysis.

## Data Availability

Anonymised transcripts are available upon reasonable request from the corresponding author.
