# Supplementary material for: Impact of Displacement on Refugee Women's Sexual and Reproductive Health: A Participatory Study Using Photovoice
Source: BJOG. 2025 Aug 19;132(13):2204–16. doi: 10.1111/1471-0528.18328 (PMC12592786; doi:10.1111/1471-0528.18328)
Supplement: Supplementary file 1 — Figure S1: Collection of photographs. [file BJO-132-2204-s002.pdf]

Annex A.

C**ollection of photographs with accompanying titles and participant texts**

Note: underlined titles show those selected for exhibition.

Photograph 1

Title: **Lucky Baby**

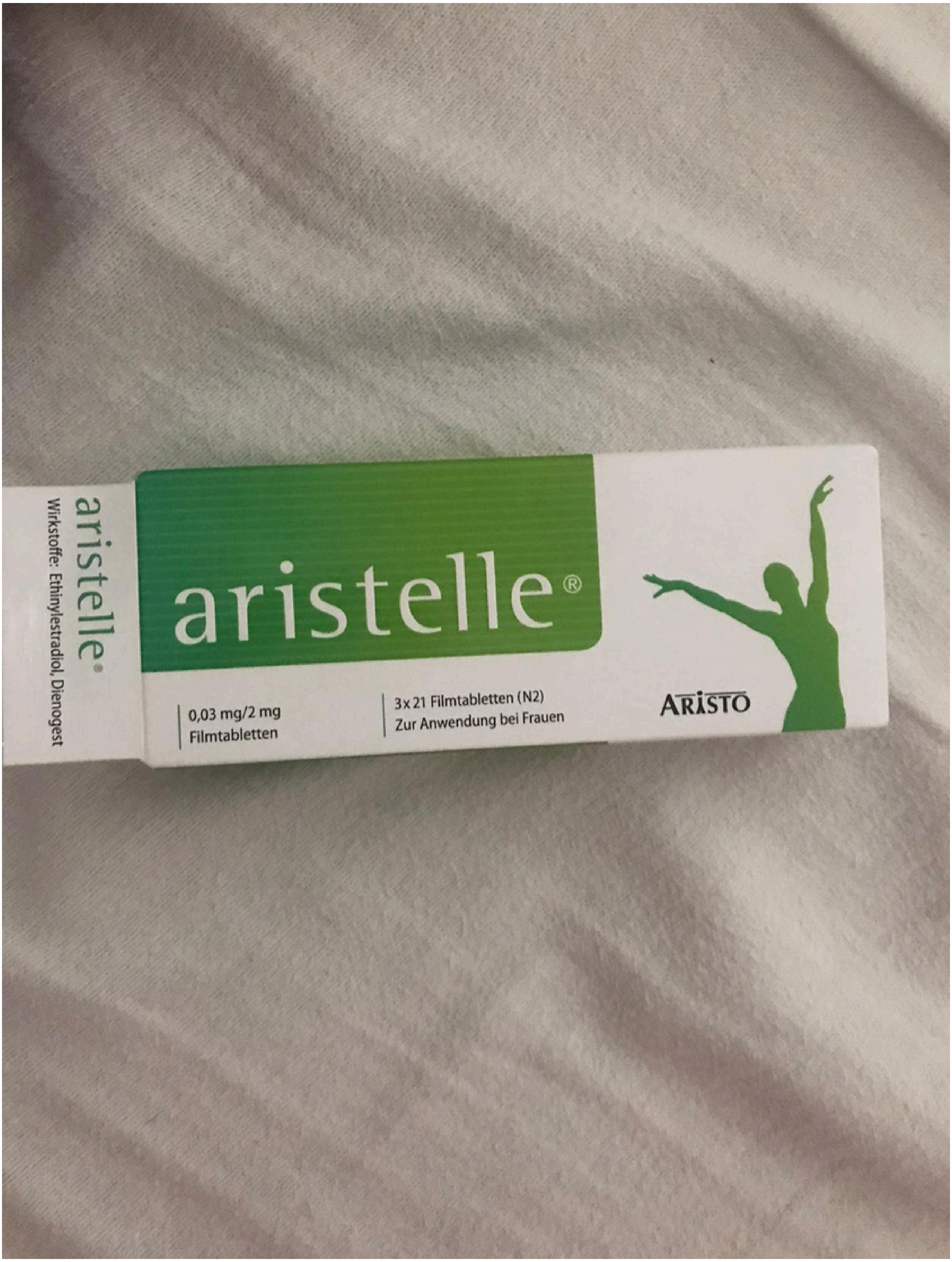

"She said are you kidding? I said: No, there's a baby! I see the placenta! I see everything because in [my home country] I was working with the ultrasound. […] I always like to learn something with the health science, and I know a little bit with the ultrasound, and I see the placenta I said I'm not kidding there is a baby! And the doctor said: yes there's a baby."

Photograph 2

Title: **Hope**

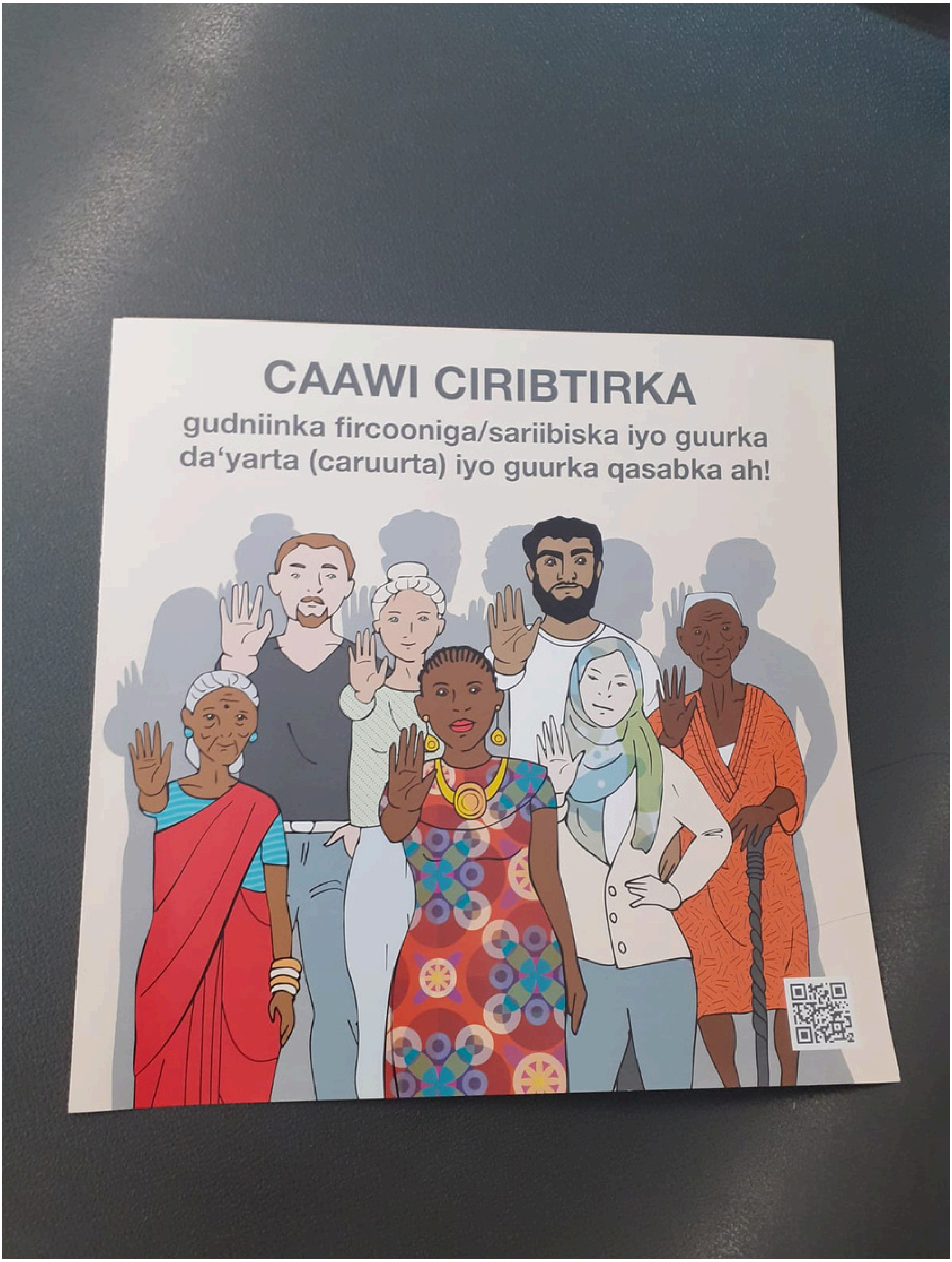

"As I said before there's a lot of struggling with the FGM. You know some people they complaining that they lost all of their clitoris or every part of their vagina so that they don't have any reconstruction or any surgery according to the FGM but now here there is a surgery like free and yeah and the government support them if they need like to make a reconstruction."

Photograph 3

Title: **Abebe**

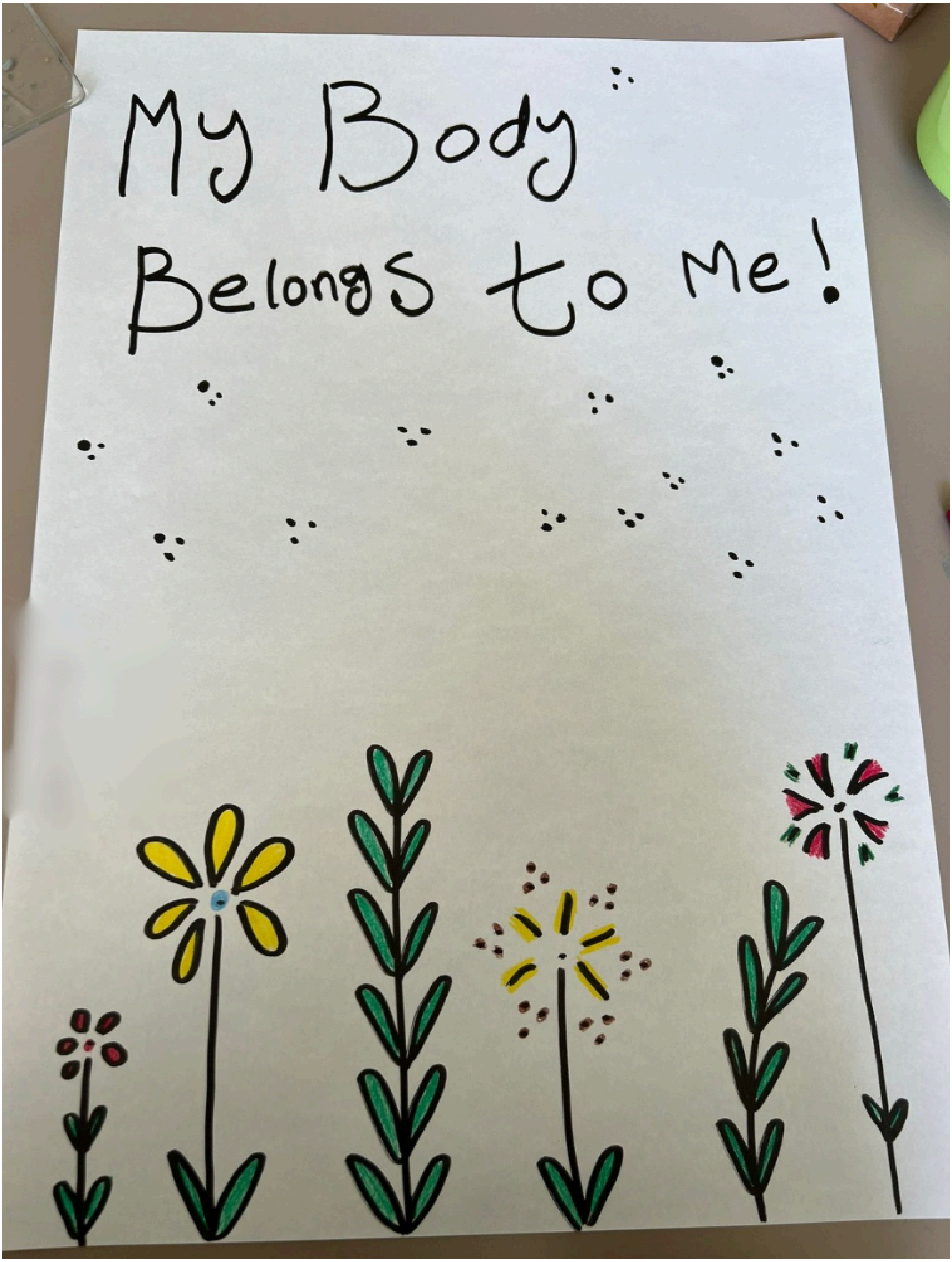

"I want to tell the parents not to do that. Like those flowers: if we keep them, our flowers could be beautiful - they can have a nice fresh air, everything."

Photograph 4

Title: **From the Studio to the Kitchen**

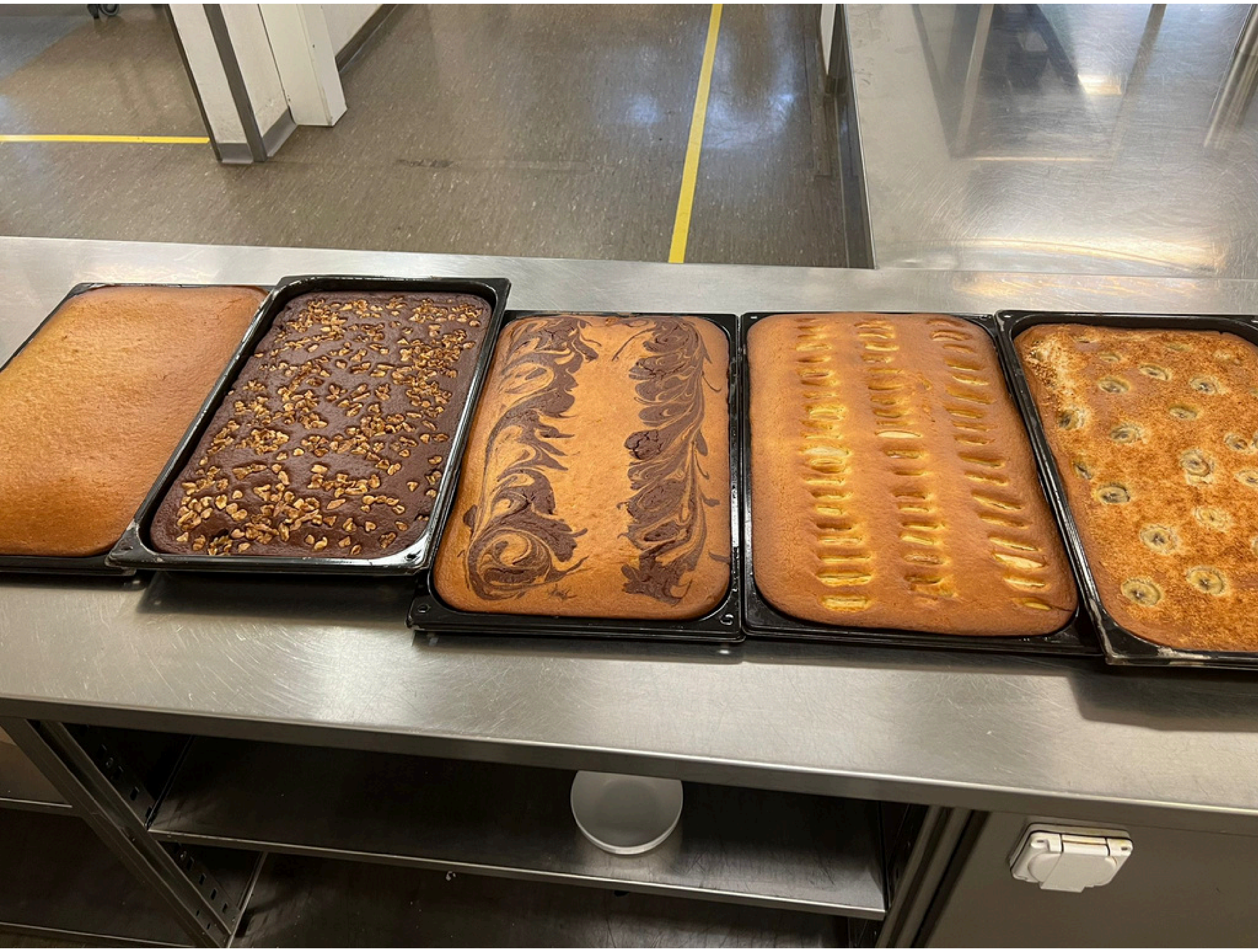

"Like after the war broke out there was not any place for me in [home country] that's why. I fight with the government they tried to kill me and I had to leave that's why. […] To be honest I think I love being journalist, […] it's not easy to accept it everything is like it's not easy. Now like I already accept it, but first it was like that place it doesn't feel comfort for me. Like going from the studio to the kitchen it doesn't give sense for me."

Photograph 5

Title: **Reversed Food Line**

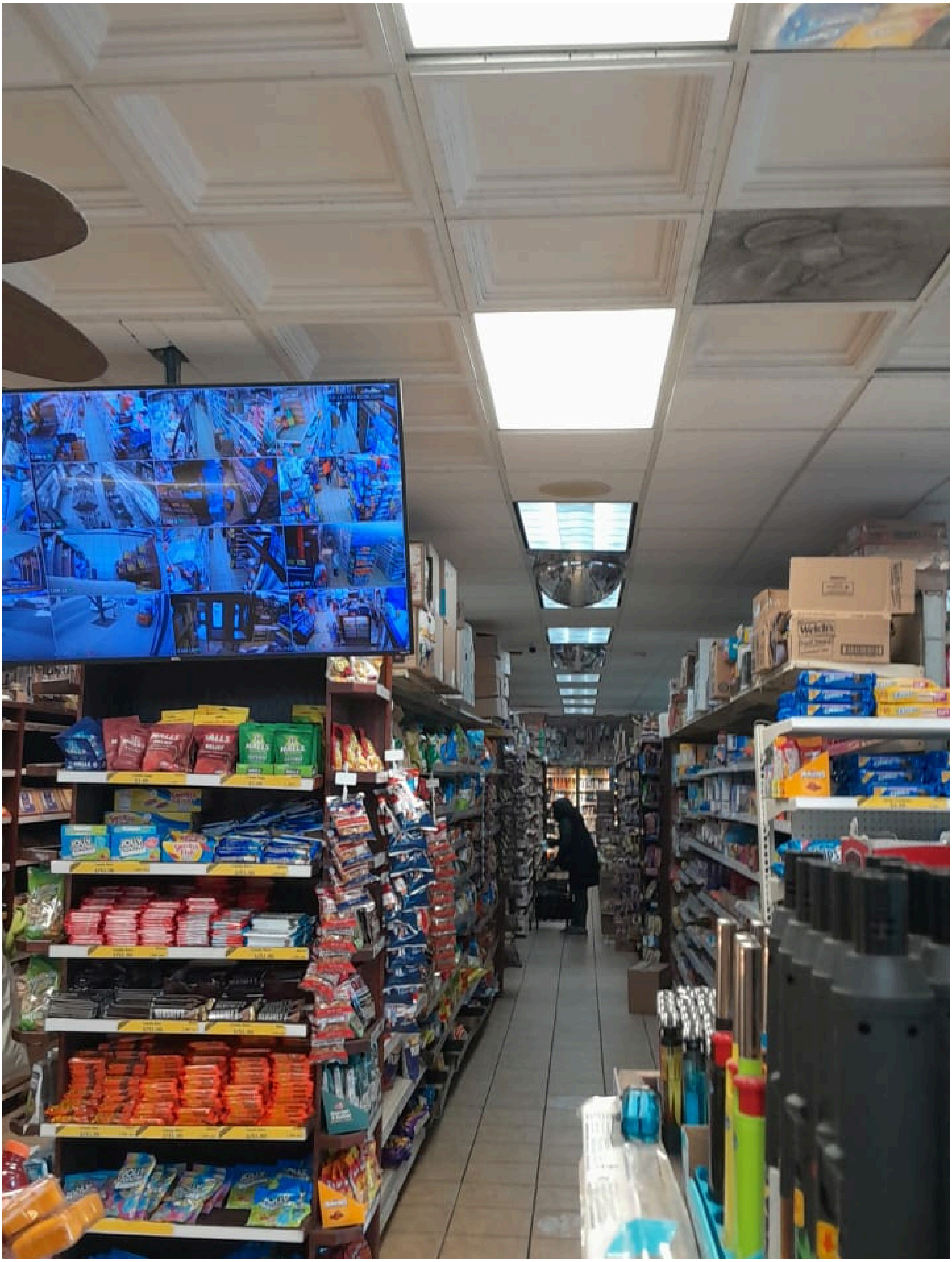

"Yeah, it's hard work… it's not my field but you know just waiting and not doing anything. It's better to just go and work."

Photograph 6

Title: **Stairs to the Toilet**

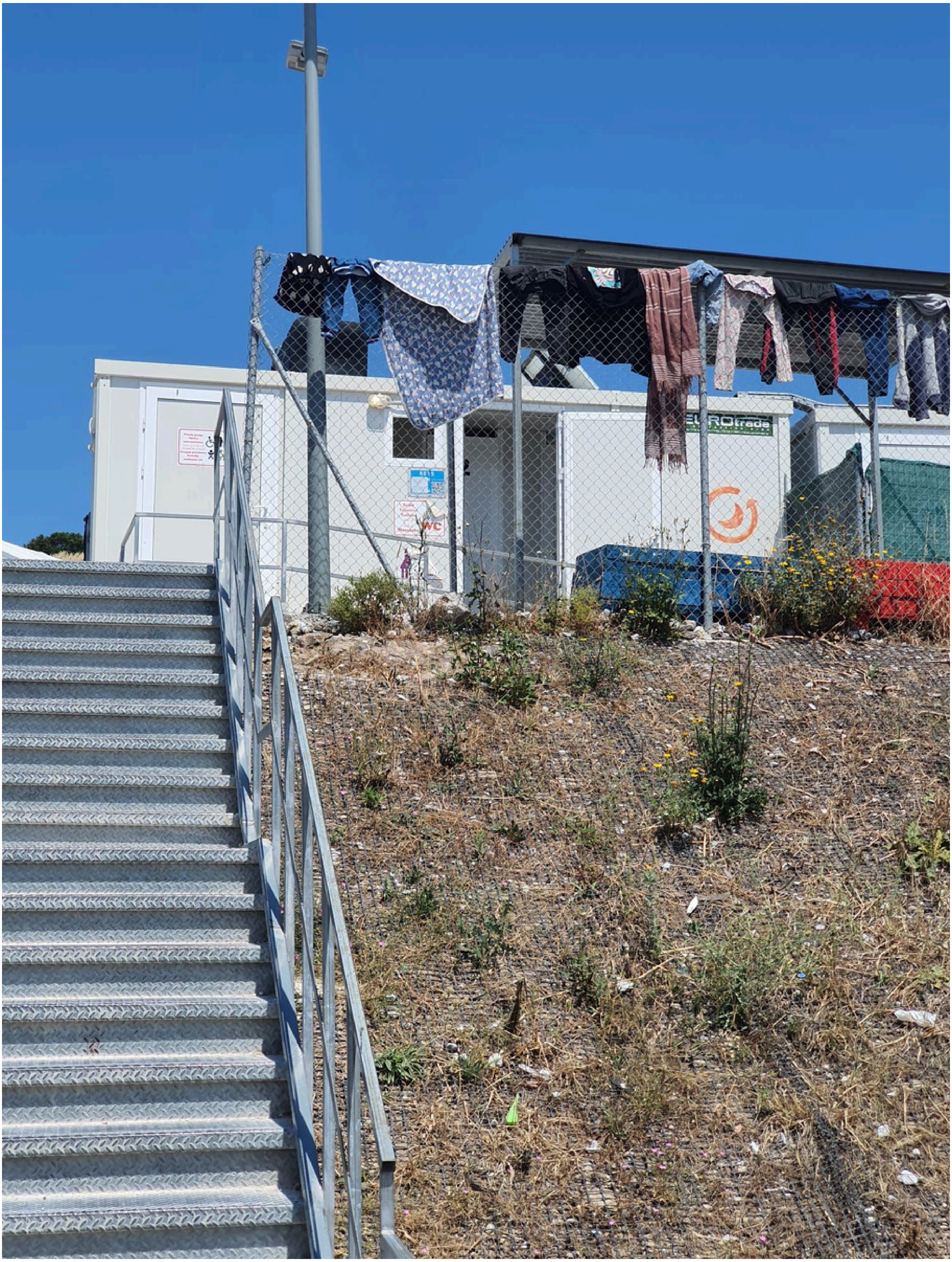

"And that's why I chose this picture. The reason that there is also stairs in this picture it's difficult for the women that they had delivery to go up from the stairs and reach the toilets and bathrooms."

Photograph 7

Title: **Green Flower**

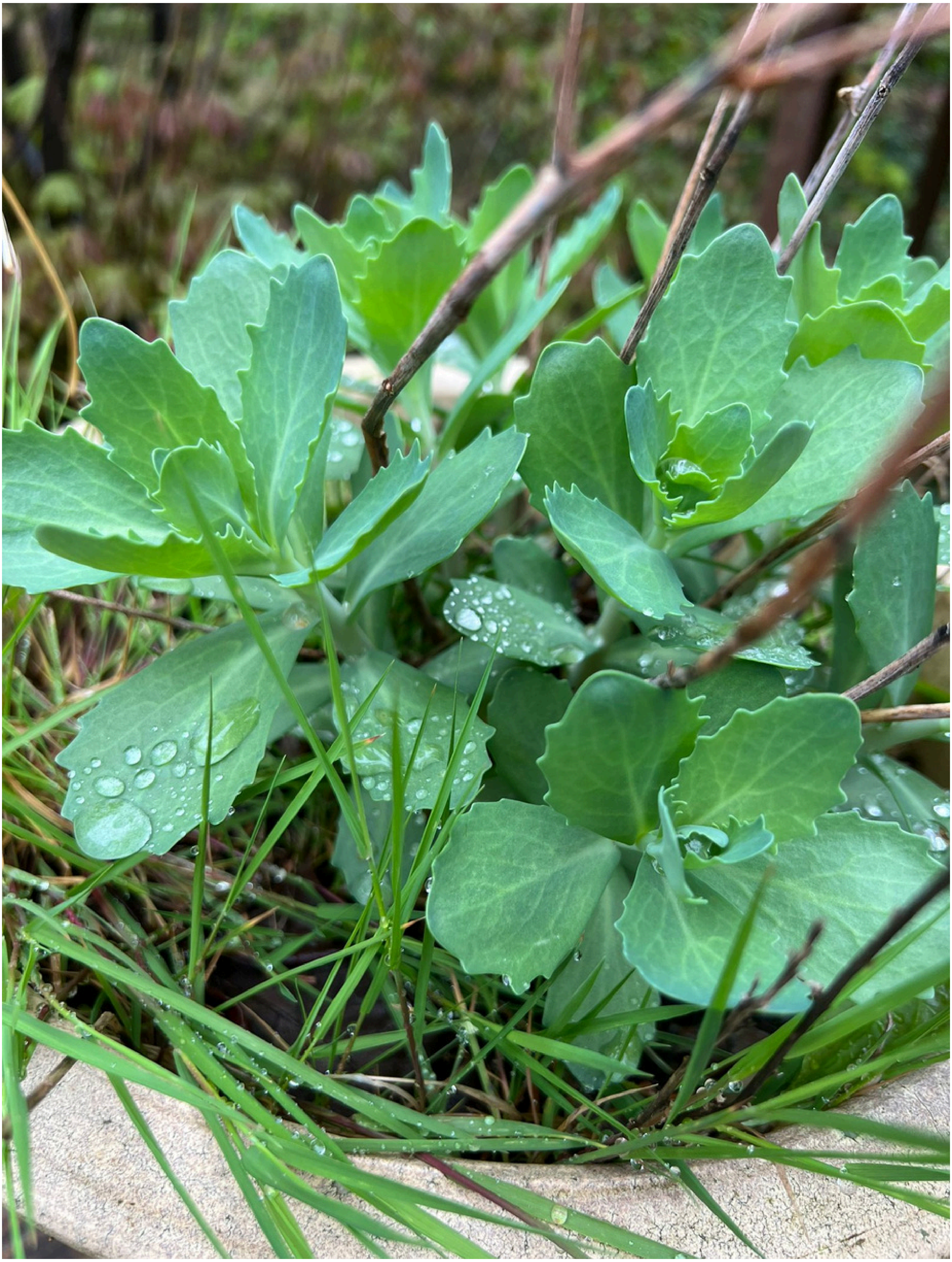

“When I see this picture it gives me good feeling. The first one with the grass and green things – like green things for me […] I found it something place like talk with yourself, having some time really to think and like to get some fresh air. […] if you want you can shout also. Like I’m stressed or something I’m going to go there and shout and then I think a time.”

Photograph 8

Title: **Instability**

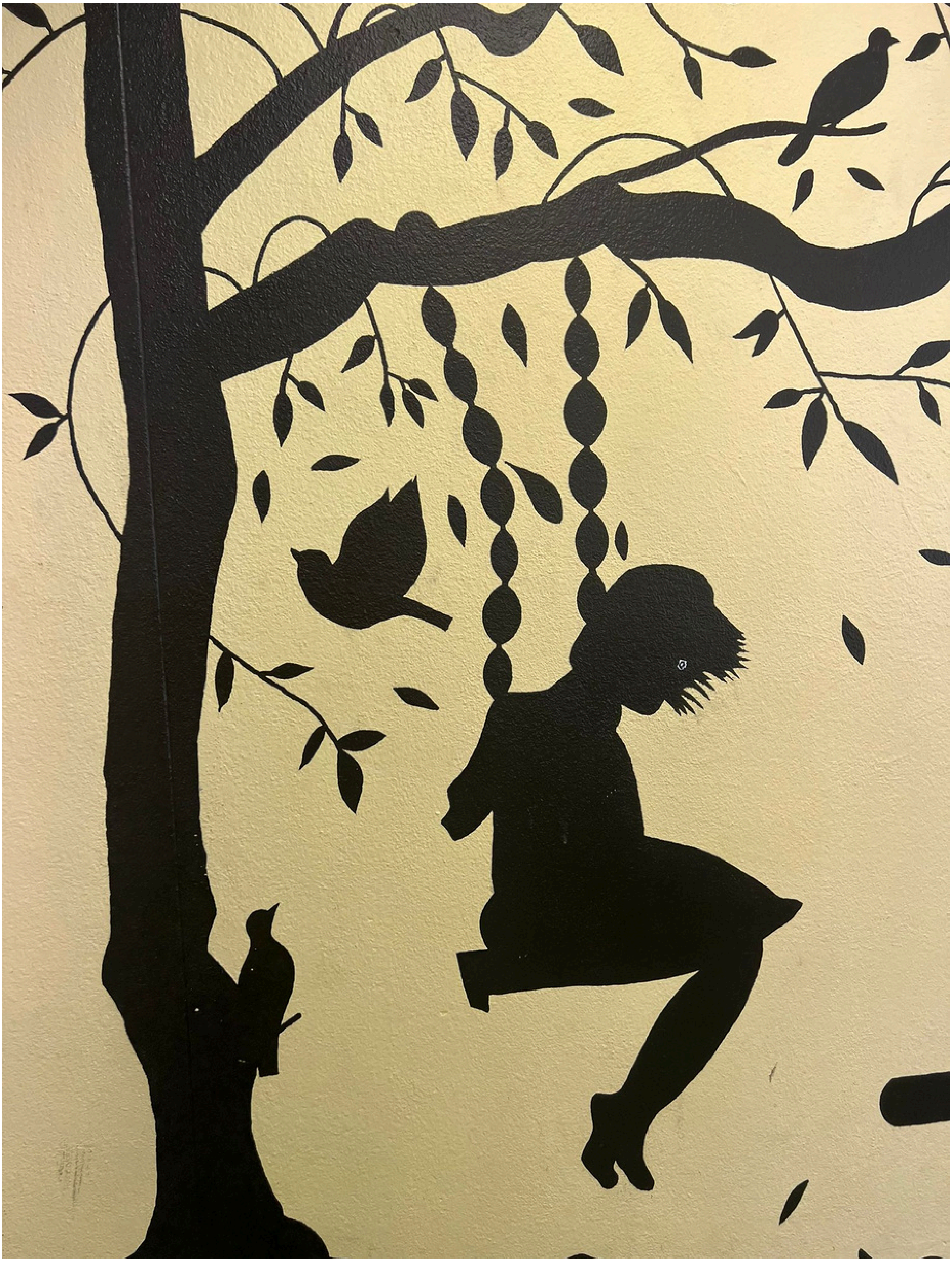

"If you want to be sad, even if you want to laugh, there is no place here. Like even if I want to be happy I cannot be happy to be honest. Even if I want to laugh, maybe someone could cry next to me, so it doesn't feel good. So if you want to, you have to control your emotions inside the house."

Photograph 9

Title: **Fuzzy Futures**

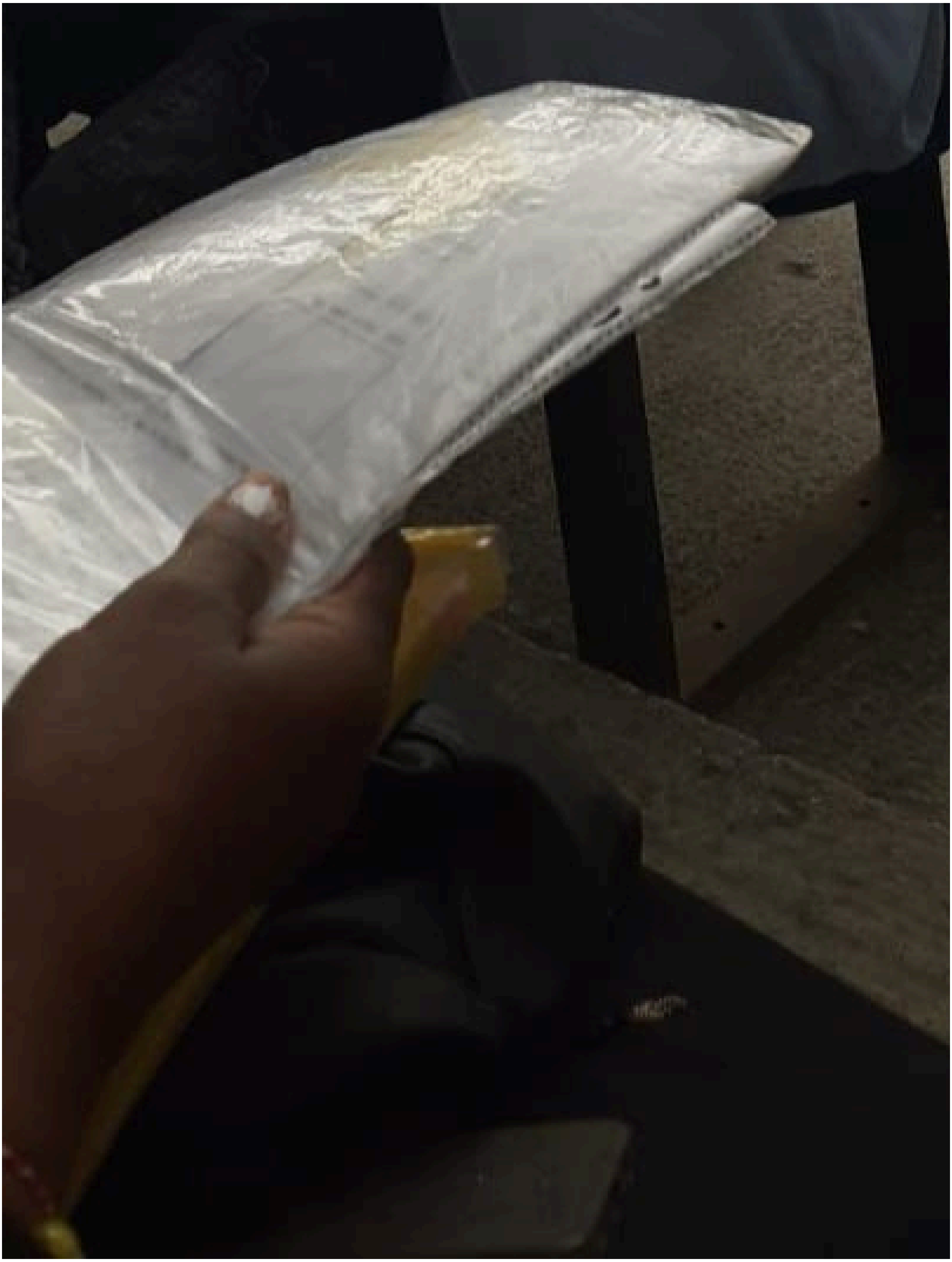

"Yes, to the instability, so because this picture is more about like waiting for the interview or for the answer and then it's instability because you don't know what's going on."

Photograph 10

Title: **The Appointment**

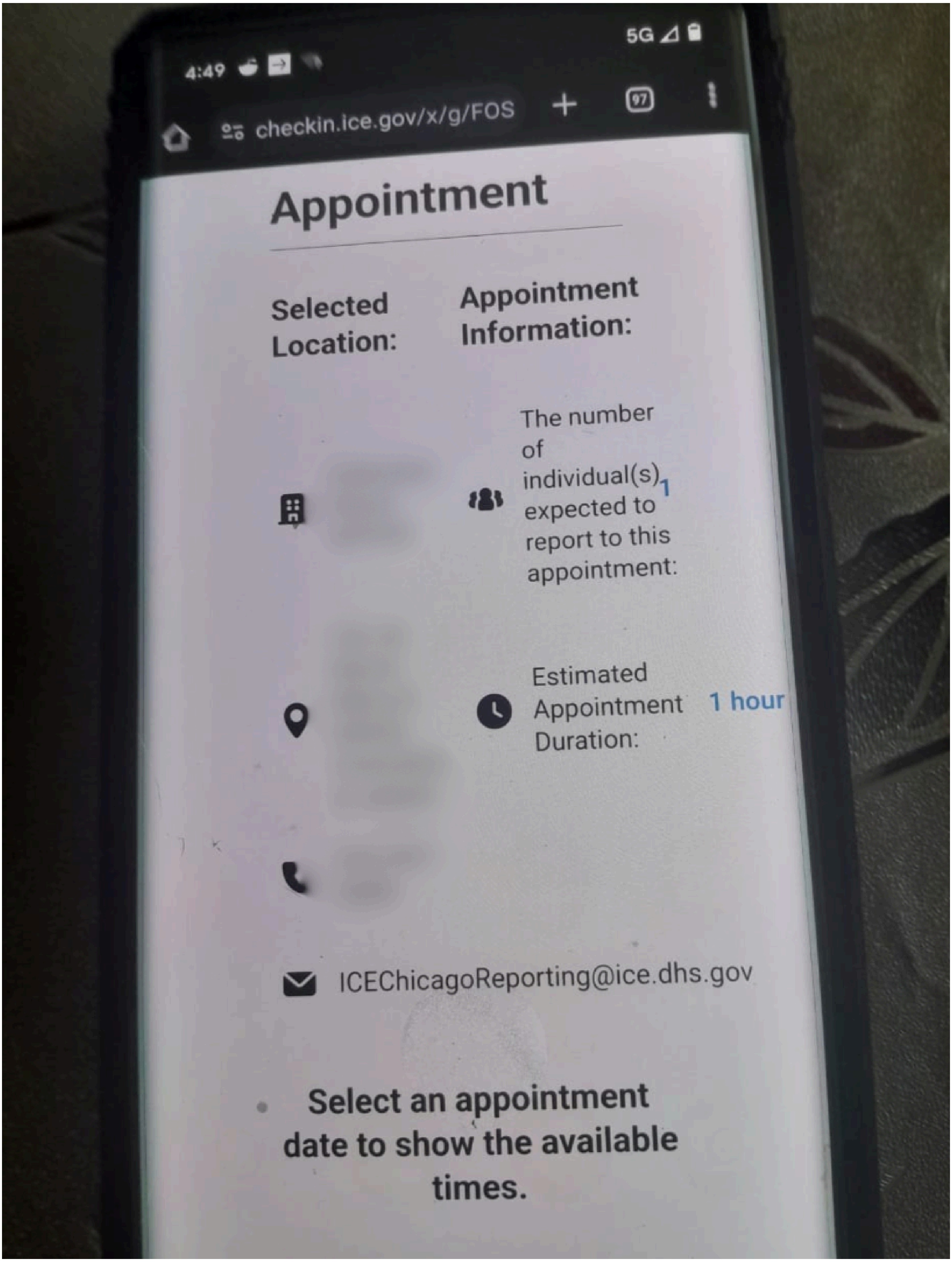

"Imagine what I get.. So they give me an appointment for 2027!"

Photograph 11

Title: **[Excluded]**

[Photograph Excluded]

"They told me that's how they scared now from the police, even when they see the police inside the camp because they have really bad memories from the Greek police. They told me how they have been checked by the male police guard even they checked their very - how can I tell - your personal areas and private parts of their body. So, because of that I sent you this picture and how this kind of memories caused some kind of mental problems for the women."

Photograph 12

Title: **Camp or Prison?**

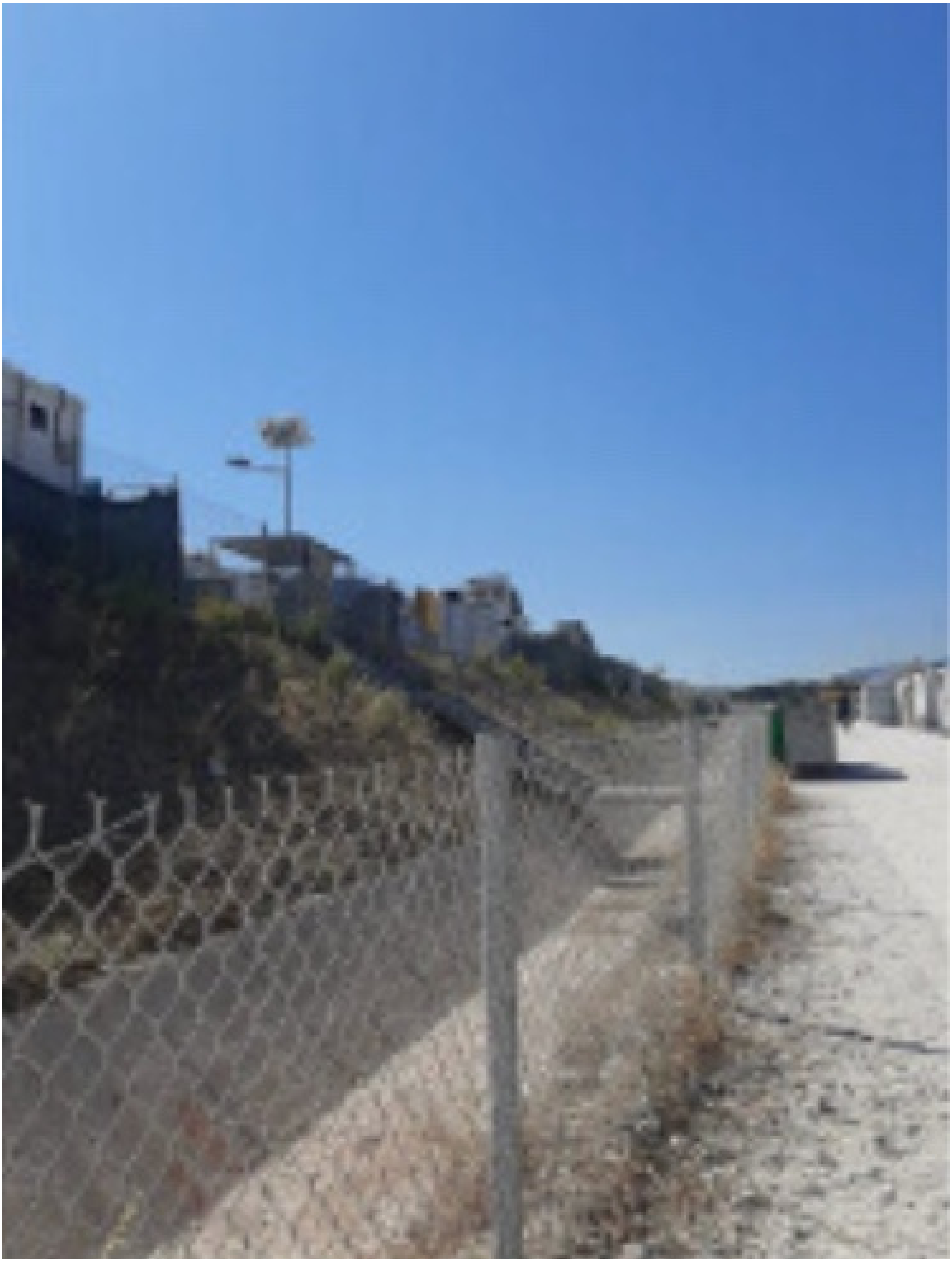

"The fence always reminds me prison. I think that when there is a fence, I remind Moria. Moria and all camps."

Photograph 13

Title: **Long Wait**

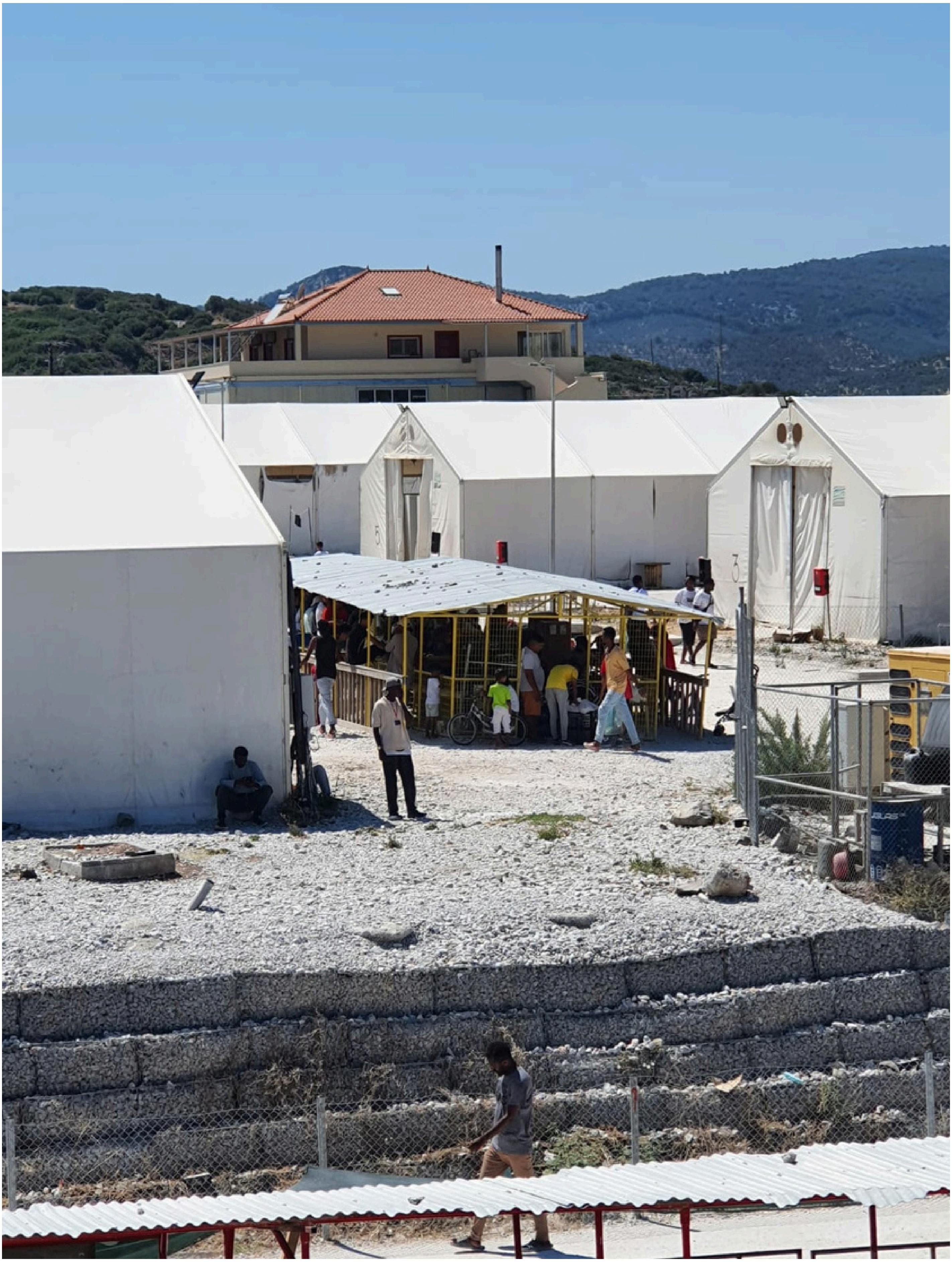

"Also the long lines. If it was today for example, if they want to visit a doctor about problem that they have and also a women's doctor for example, again gynecologist or something like this, they have to wait in the long lines for so many others and also maybe so many weeks and days until they can have an appointment with this kind of doctors."

Photograph 14

Title: **Injustice**

[Photograph Excluded]

"Even though you're in a house it still feels like a tent"

Photograph 15

Title: **The Ticket**

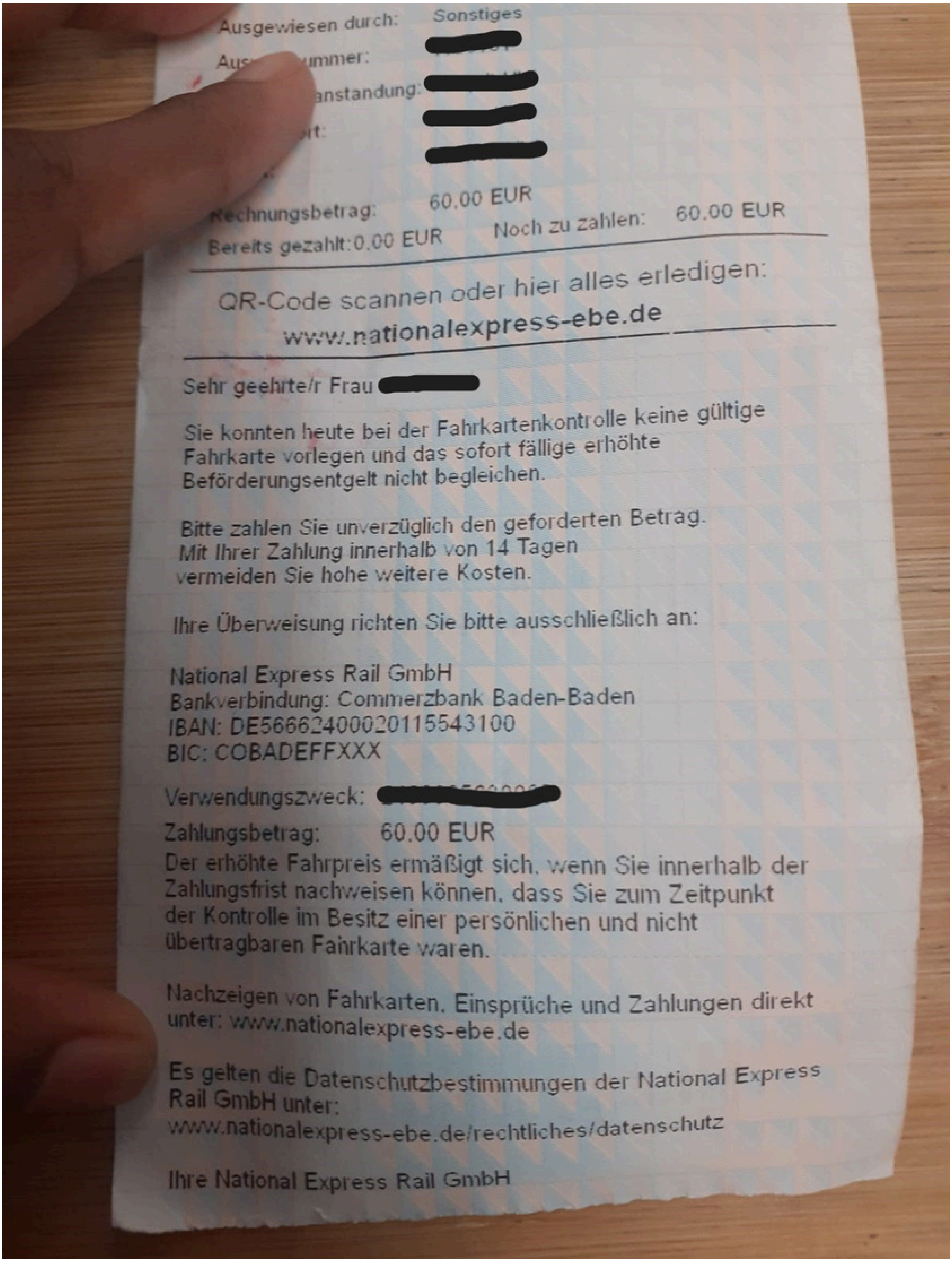

Ausgewiesen durch: Sonstiges

Aus[illegible]ummer: [redacted]

[illegible]anstandung: [redacted]

[illegible]rt: [redacted]

[illegible] [redacted]

Rechnungsbetrag: 60.00 EUR

Bereits gezahlt:0.00 EUR Noch zu zahlen: 60.00 EUR

QR-Code scannen oder hier alles erledigen:

www.nationalexpress-ebe.de

Sehr geehrte/r Frau [redacted]

Sie konnten heute bei der Fahrkartenkontrolle keine gültige Fahrkarte vorlegen und das sofort fällige erhöhte Beförderungsentgelt nicht begleichen.

Bitte zahlen Sie unverzüglich den geforderten Betrag. Mit Ihrer Zahlung innerhalb von 14 Tagen vermeiden Sie hohe weitere Kosten.

Ihre Überweisung richten Sie bitte ausschließlich an:

National Express Rail GmbH
Bankverbindung: Commerzbank Baden-Baden
IBAN: DE56662400020115543100
BIC: COBADEFFXXX

Verwendungszweck: [redacted]

Zahlungsbetrag: 60.00 EUR

Der erhöhte Fahrpreis ermäßigt sich, wenn Sie innerhalb der Zahlungsfrist nachweisen können, dass Sie zum Zeitpunkt der Kontrolle im Besitz einer persönlichen und nicht übertragbaren Fahrkarte waren.

Nachzeigen von Fahrkarten, Einsprüche und Zahlungen direkt unter: www.nationalexpress-ebe.de

Es gelten die Datenschutzbestimmungen der National Express Rail GmbH unter:
www.nationalexpress-ebe.de/rechtliches/datenschutz

Ihre National Express Rail GmbH

“...And then there was another control we get it out and then we stand, we have to wait another train for like 40 minutes or so. I can't imagine you know? And it was my period the first day of my period. You know I get like tight pain because of the cold, because of the stress, I can’t imagine it was really stressful.”

Photograph 16

Title: **Welcome to the Emergency Department**

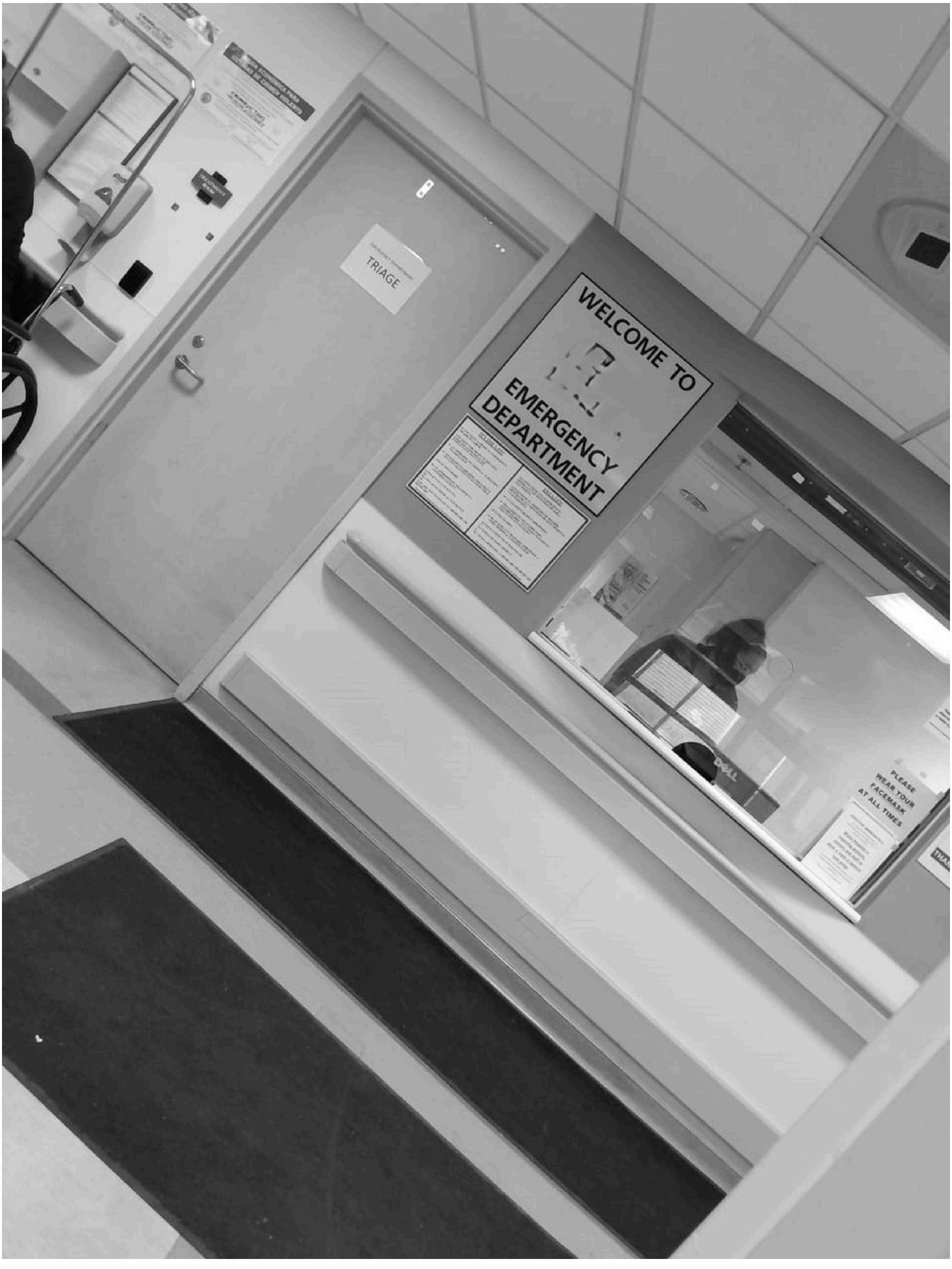

"Also when you receive a negative answer then you don't have any insurance anymore and then for some of the problems if you need to visit a doctor in hospital then you can't go there because you don't have insurance. [This is] one of the other barriers that women cannot access the good sexual and productive healthcare."

Photograph 17

Title: **Waiting for Care**

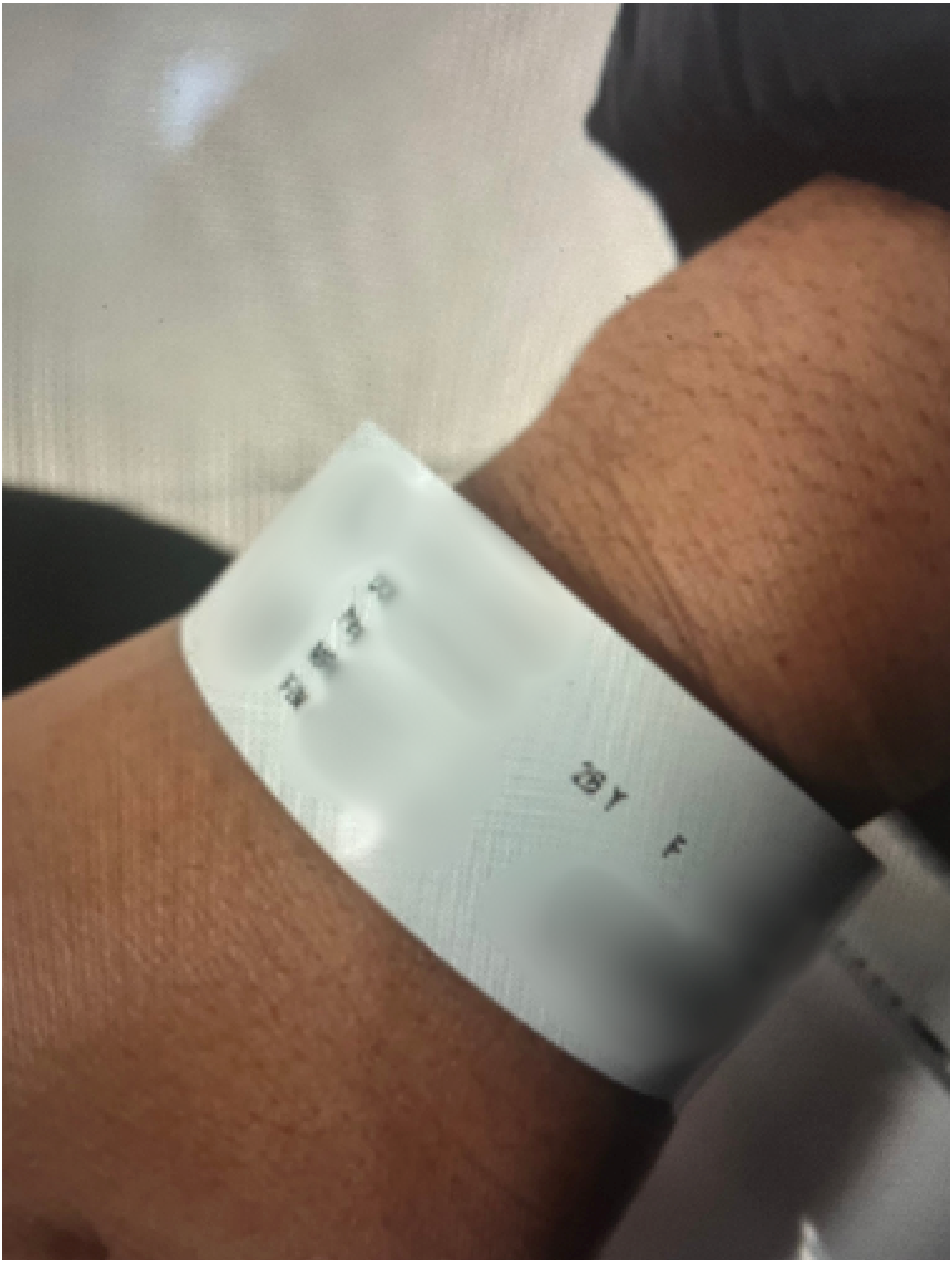

"Yes, I have to give the first priority for my health. But in this moment I can't because the first thing is I have to give the priority for my work."

Photograph 18

Title: **Kind to Everyone**

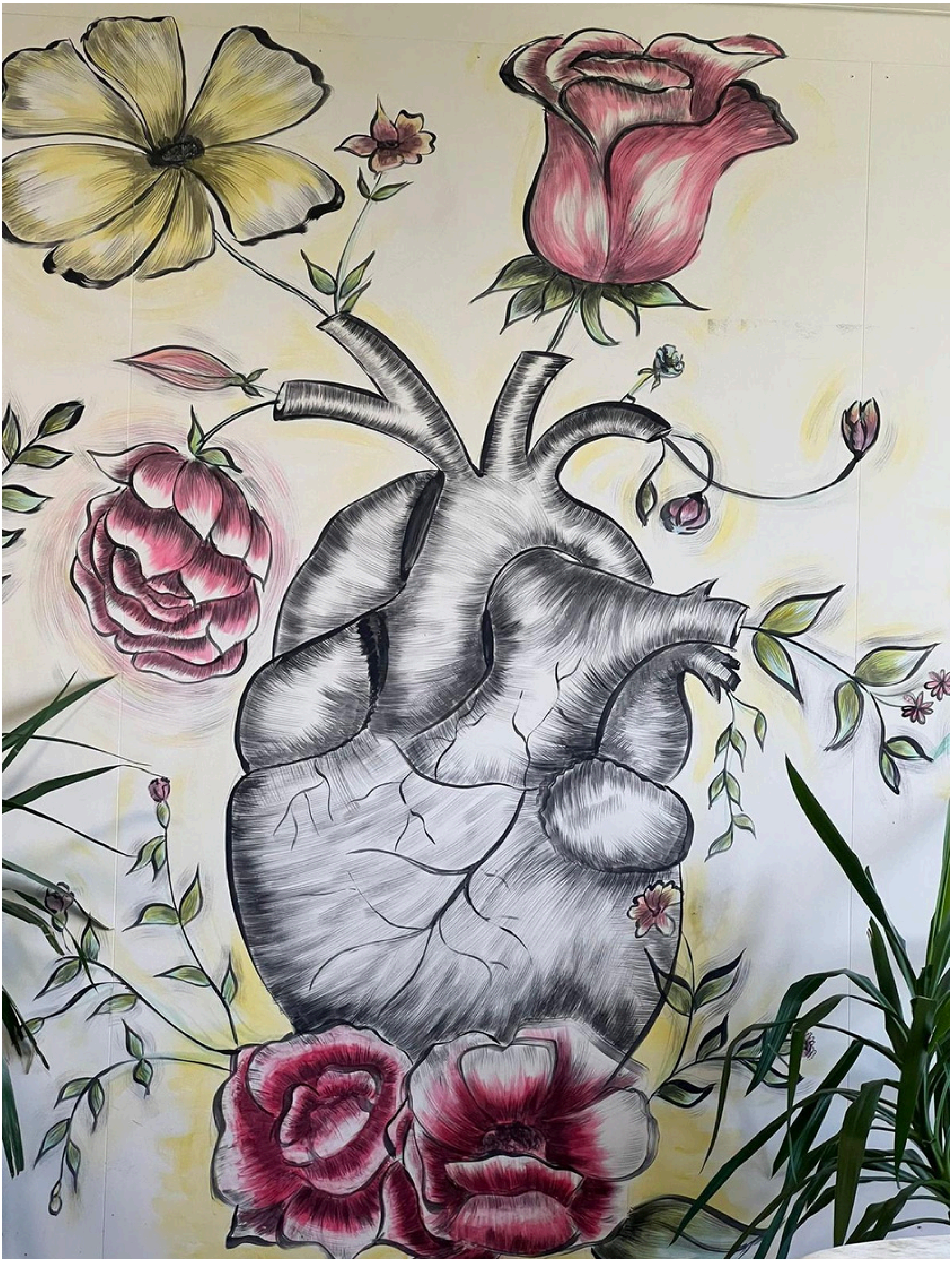

“Yeah, but when I see this heart and let’s say in this camp there is a lot of ethnicities, like a lot of people from the whole world. We are different like those flowers but at least even when we disagree each other we can be kind to each other, right? Yeah, that symbolism comes to my mind. We can be kind to everybody, we can give flowers. You cannot lose anything by being kind.”

Photograph 19

Title: **In the Dentist's Chair**

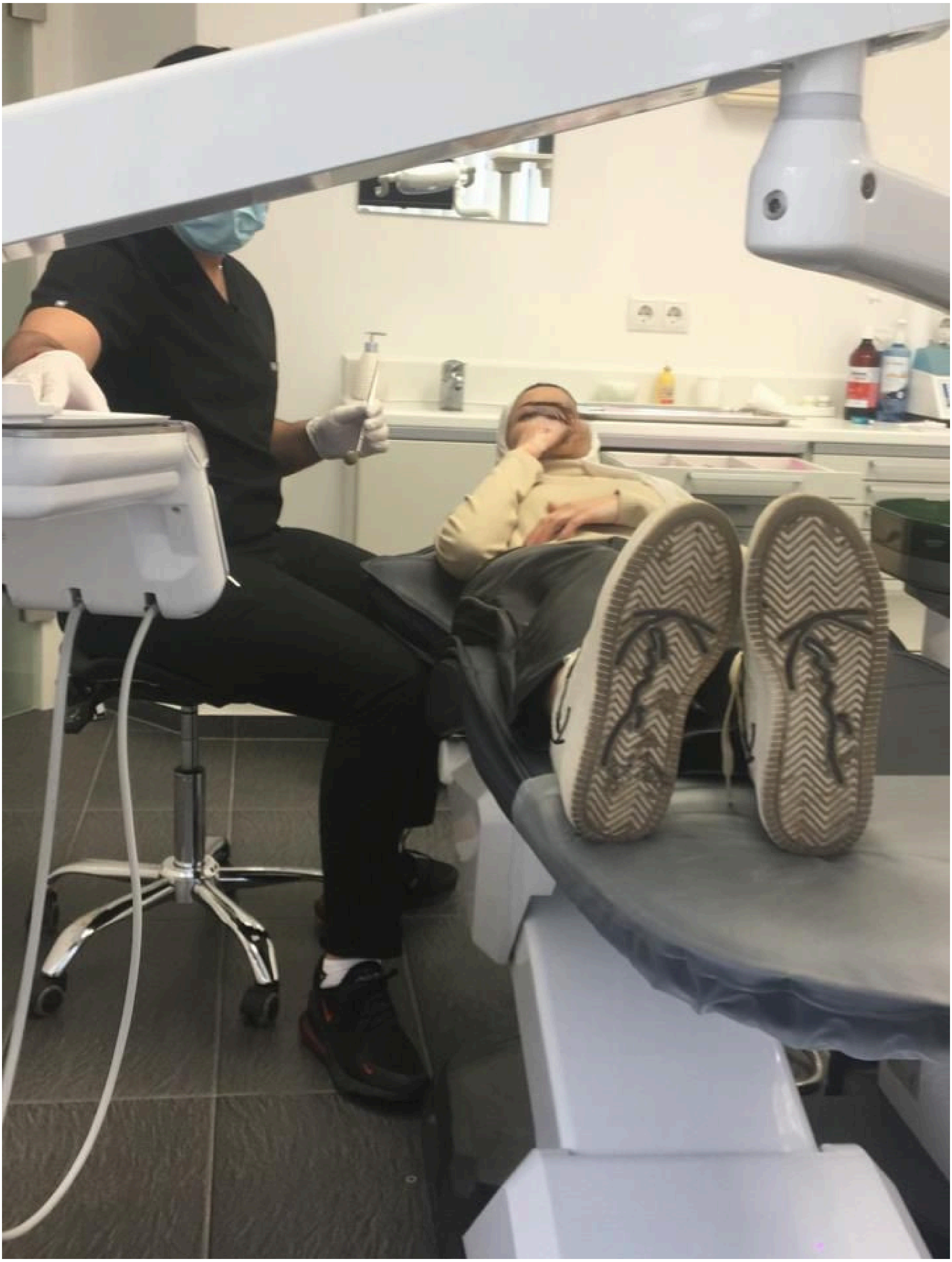

"She had to go to the dentist to get a tooth removed and it brought her a lot of comfort because she felt like she was kind of settling in by being able to go do this."

<u>Photograph 20</u>

Title: **Home-sick**

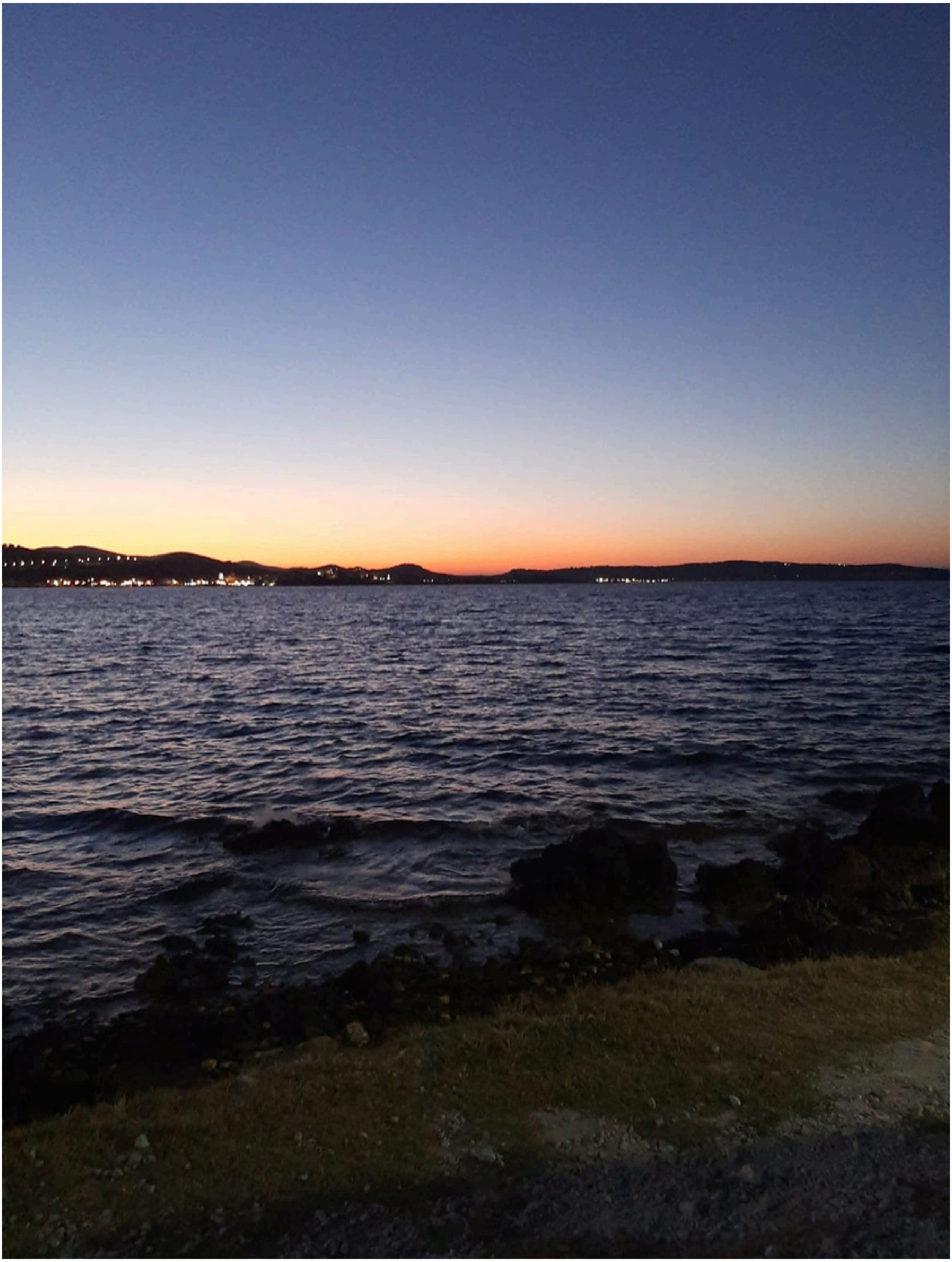

“In my mind it's like this: the sunsets and the sky is beautiful but in the contrast the sea can die the people. Even though the sea can make you feel calm and can give the people peace, but on the other hand it can be very dangerous and be like a murderer”

Photograph 21

Title: **To Support Me**

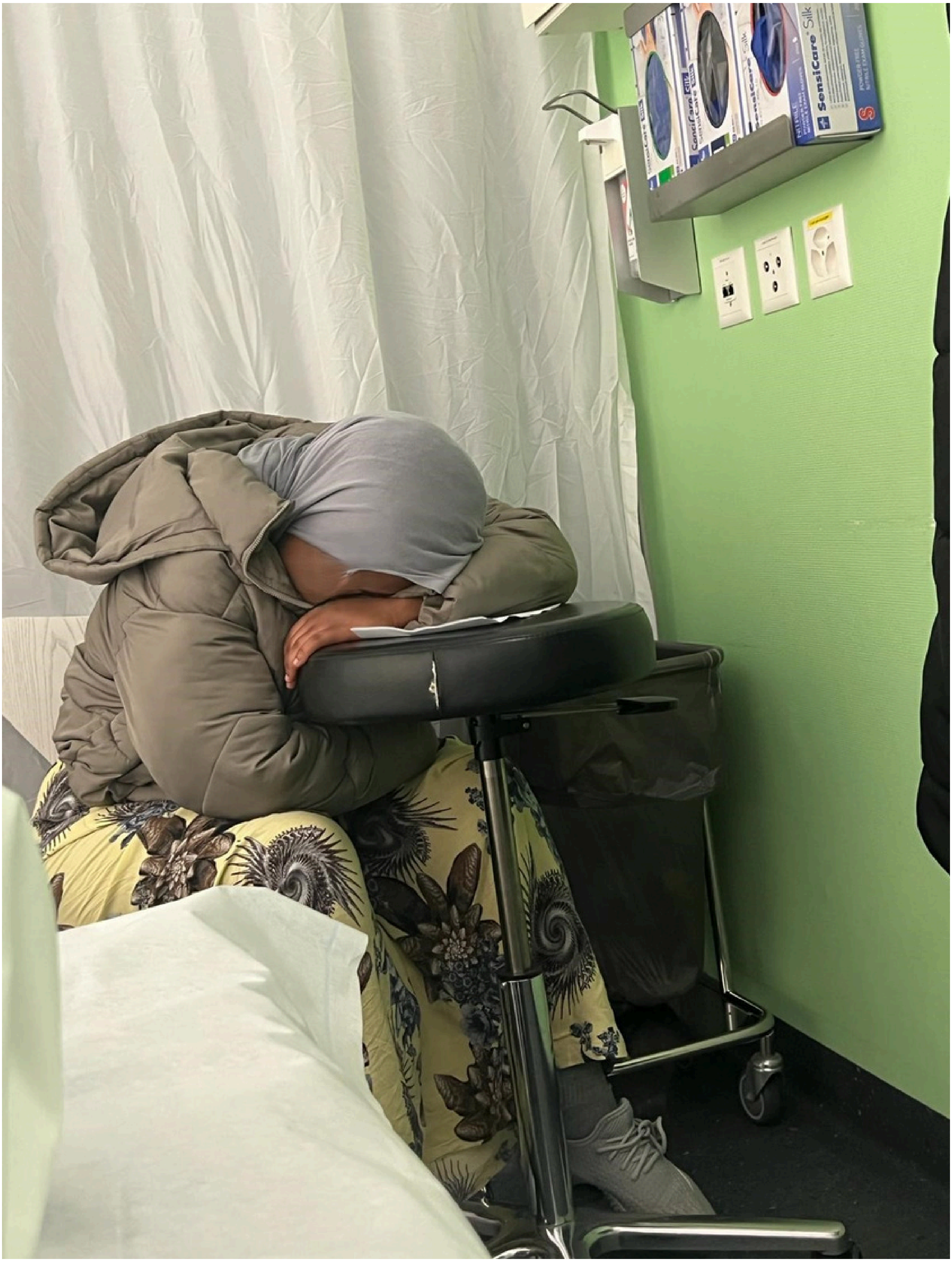

"Oh yeah that picture I take it myself. Like my phone was with me it was the second day or third day? And then my friend – she's so kind – she couldn't sleep. I don't know how say, she always keep an eye for me. She don't even want me to sleep. Like if you sleep you're going to die. And then at one moment when we were talking and she fell asleep and I take that picture."

Photograph 22

Title: **Fenced Pasts**

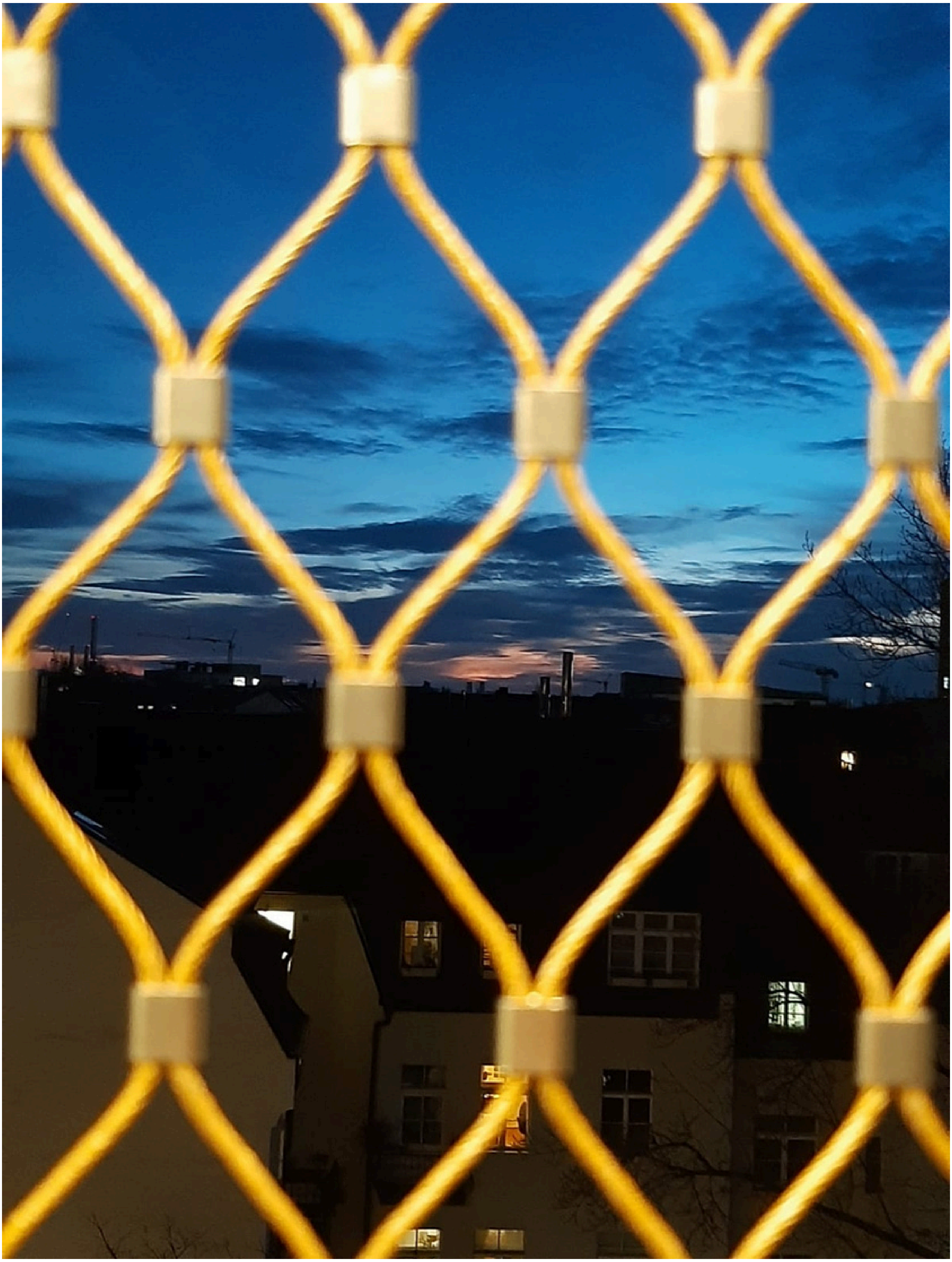

“Most of the time when I see the sunset time I miss… I miss my family more than other times. […] It’s so beautiful but then when I see it, it makes me more sad than happiness”

Photograph 23

Title: **Waiting**

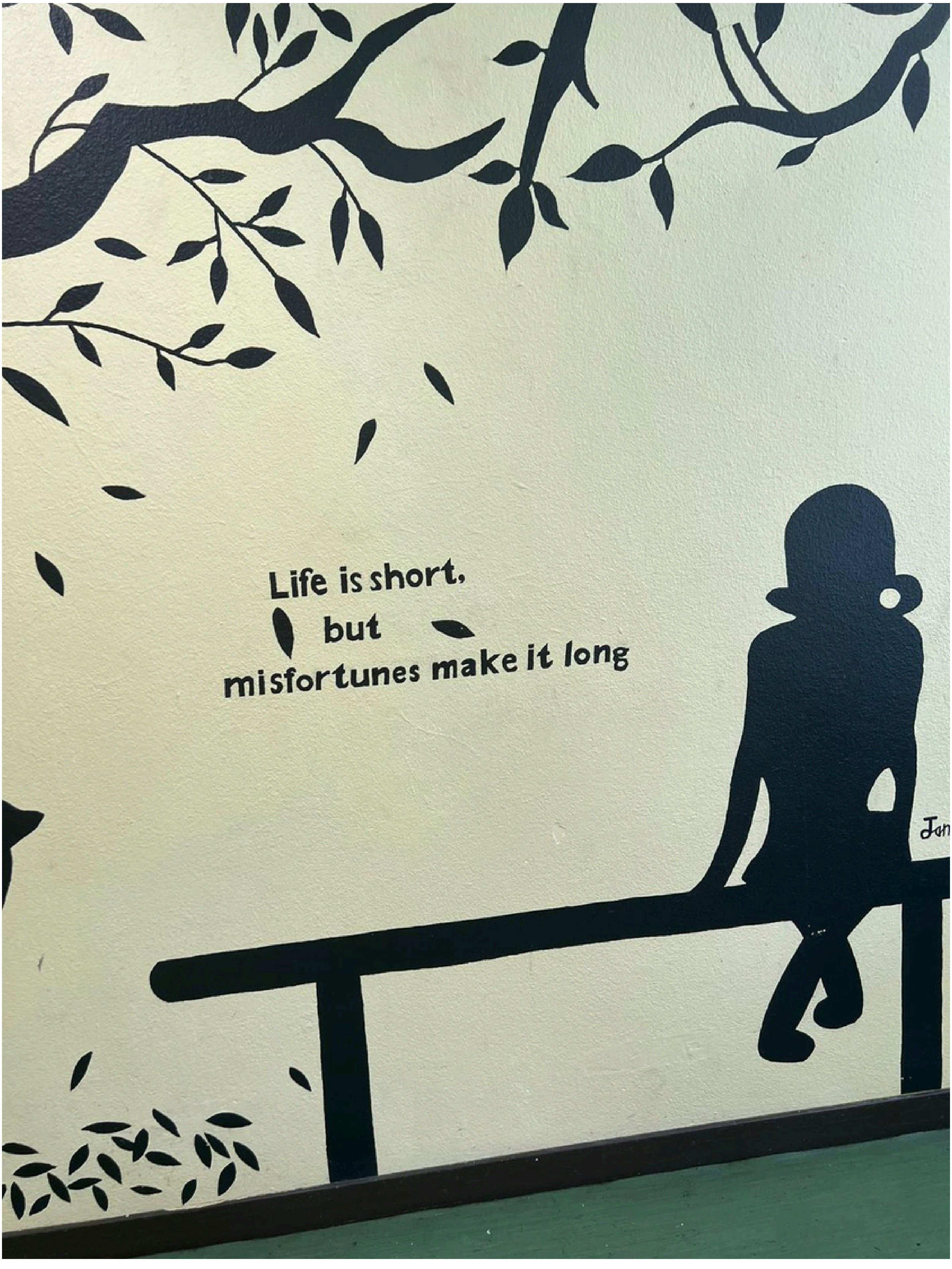

"You can see all the time like especially in the morning I can see a lot of people feeling sad and feeling fear when they have appointment with their lawyer to tell them the answer. Yeah, every morning there are a lot of people with sad faces."

Photograph 24

Title: **No Boundaries**

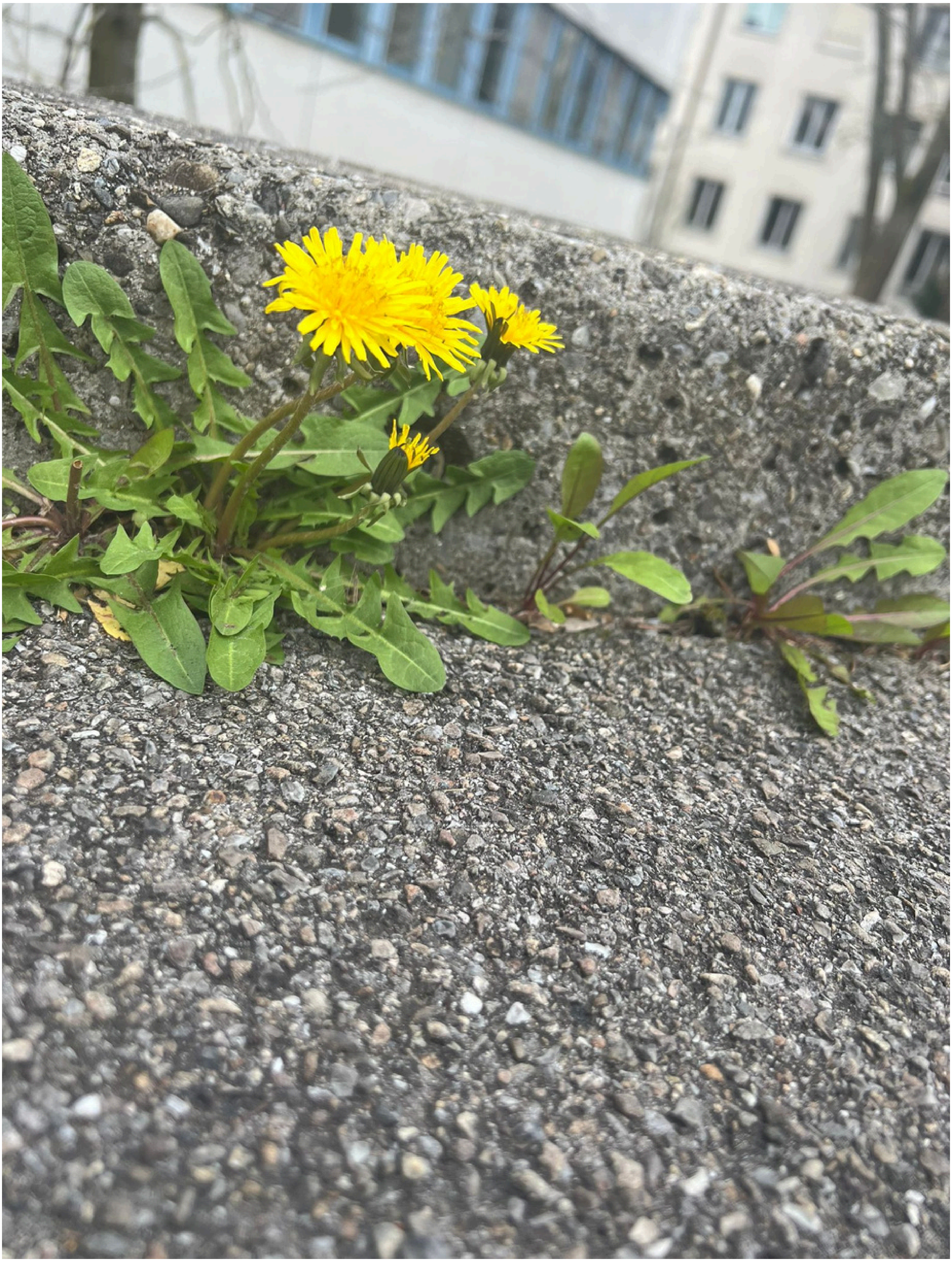

"Like these flowers. In the world there is no space for flowers, but it's just rising."

Photograph 25

Title: **The Key**

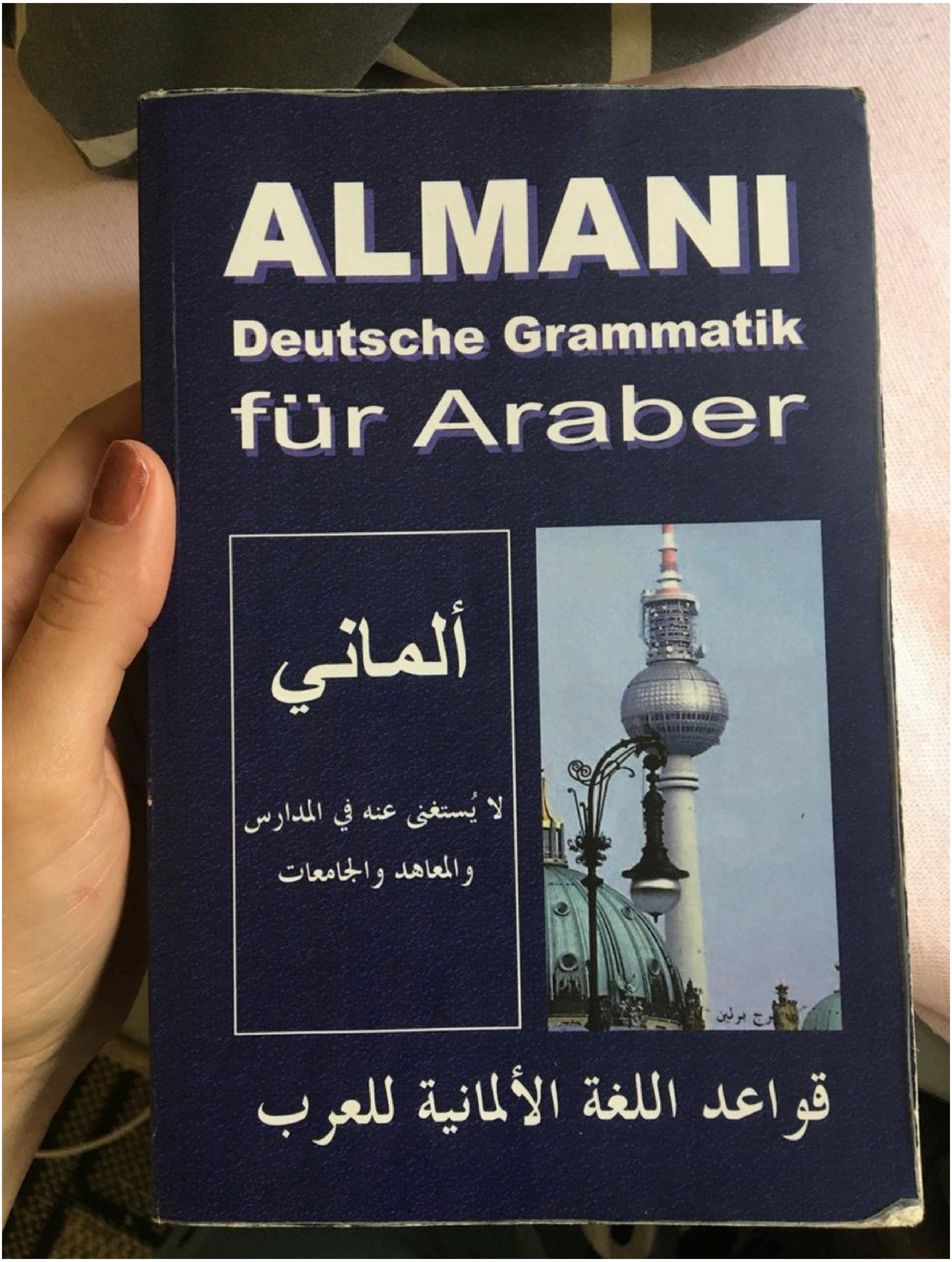

"Knowing the language is key to being part of the community"

Photograph 26

Title: **New Year**

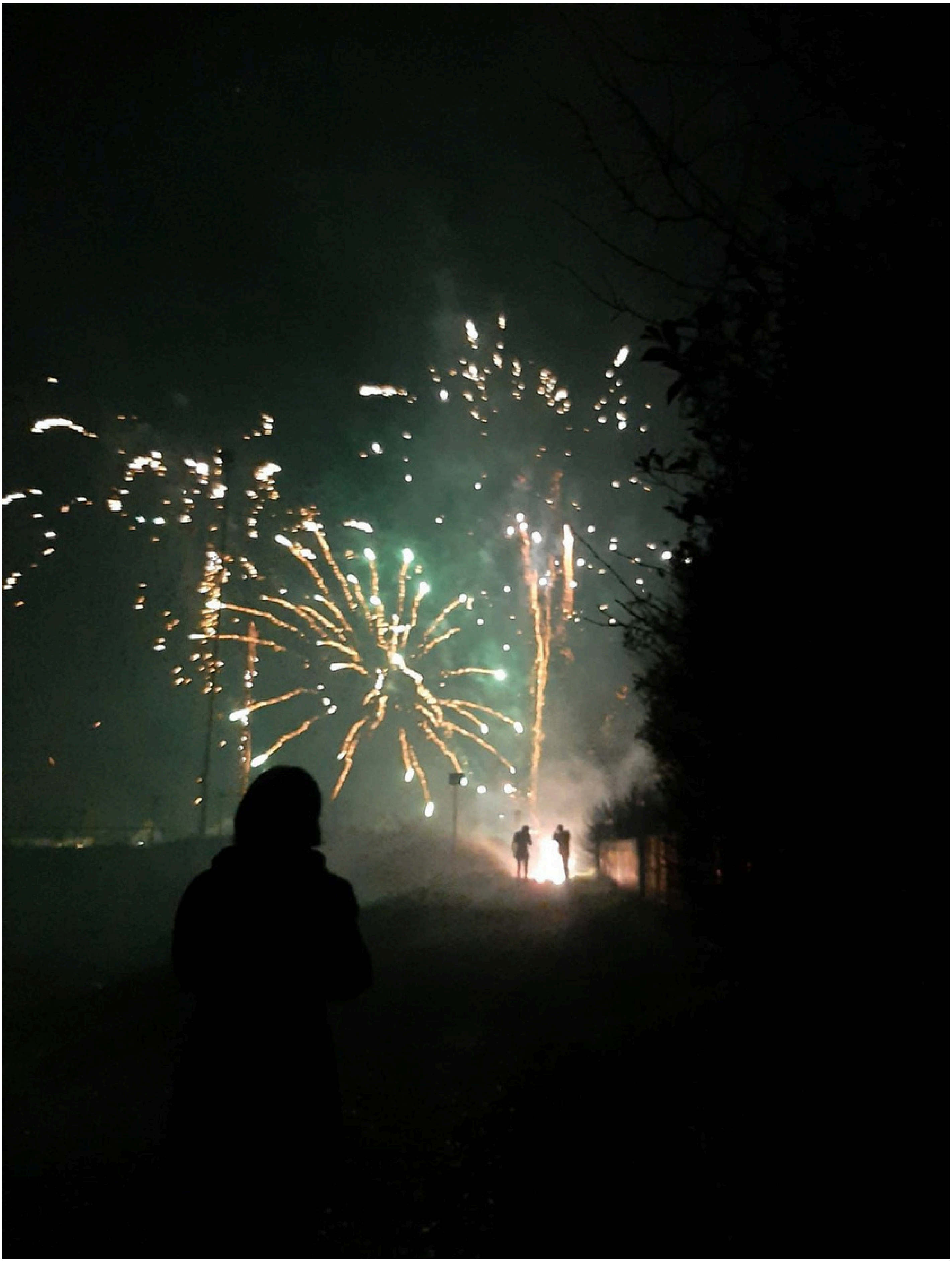

"This was our new year in Germany. Now it means home for us as well. So, since we came to Germany, we celebrate the new year here with some of our friends. It's like growing in your new home. Having a new home after being on the move for so many years."

<u>Photograph 27</u>

Title: **Celebrations**

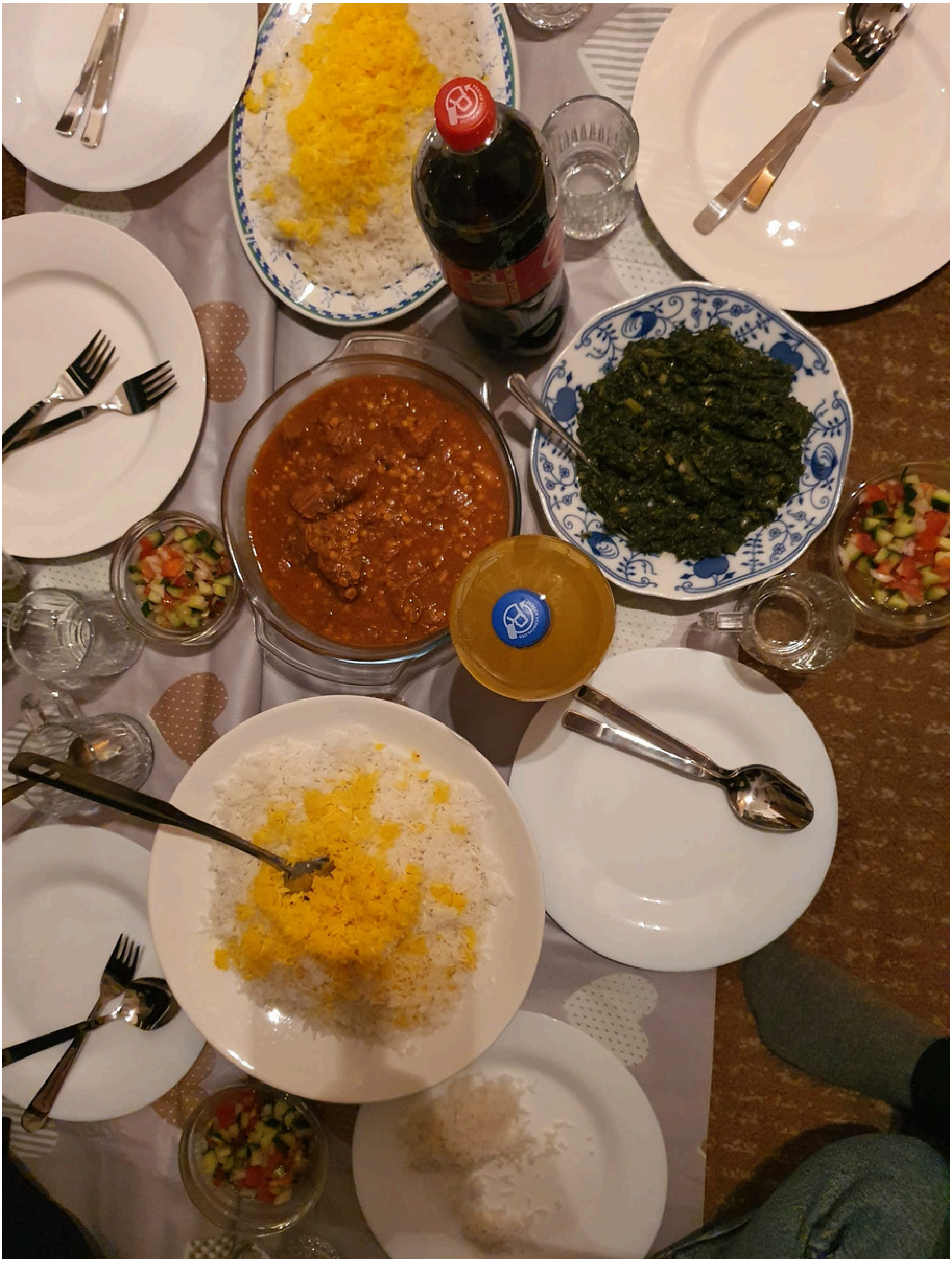

"I took it last night because it's after our C1 test and we were too busy busy busy and tired, and I was relaxed completely, and I took this picture, and I was happy."

Photograph 28

Title: **Recognition**

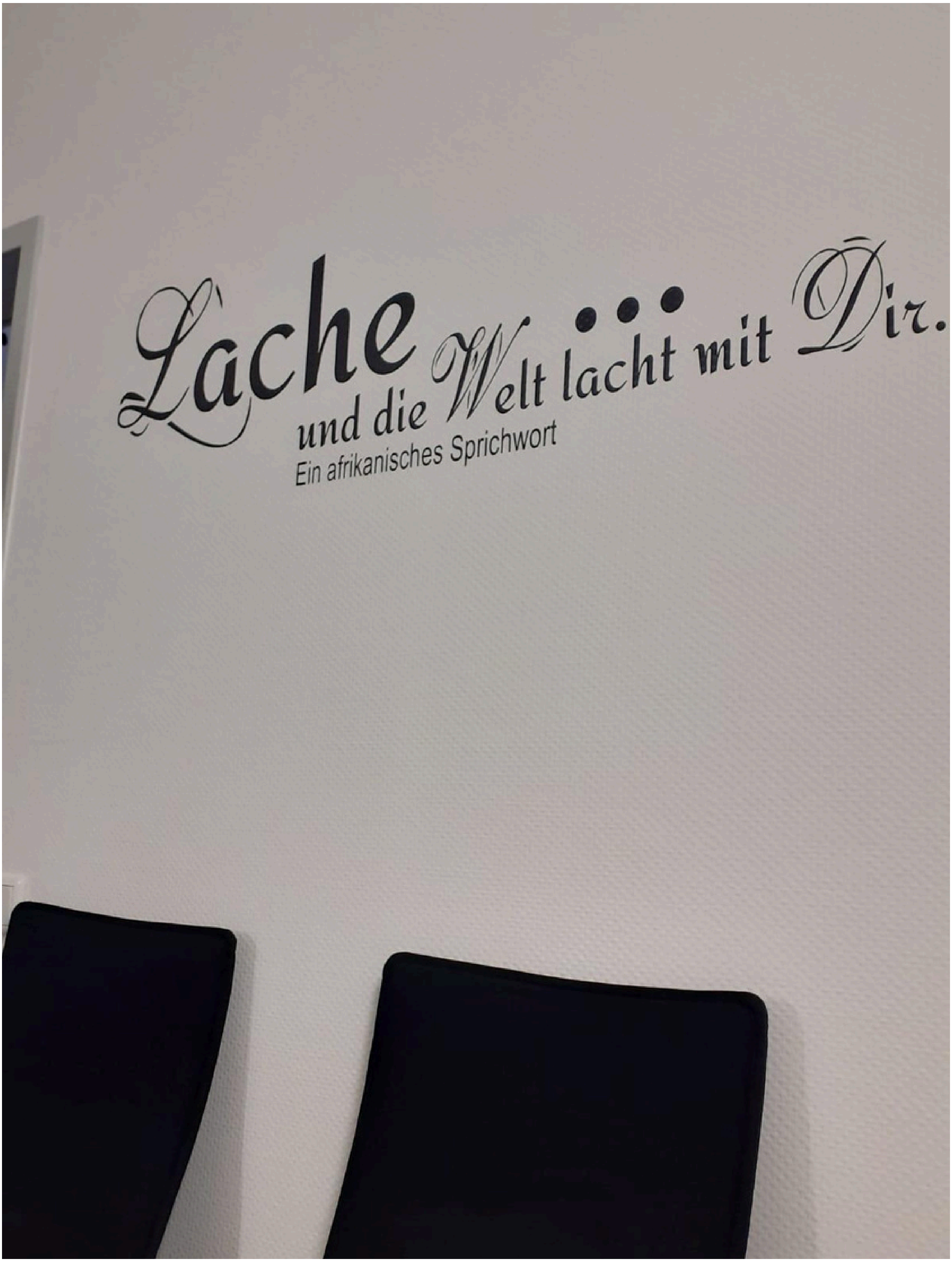

"You know for me I didn't know this Deutch at first, but when I go to hospital, the big hospital, and when I was there I see African [...] and then I translate, I see that there's an African proverb, it says laugh and the world laughs with you, so I feel connection that situation."

Photograph 29

Title: **New Skills**

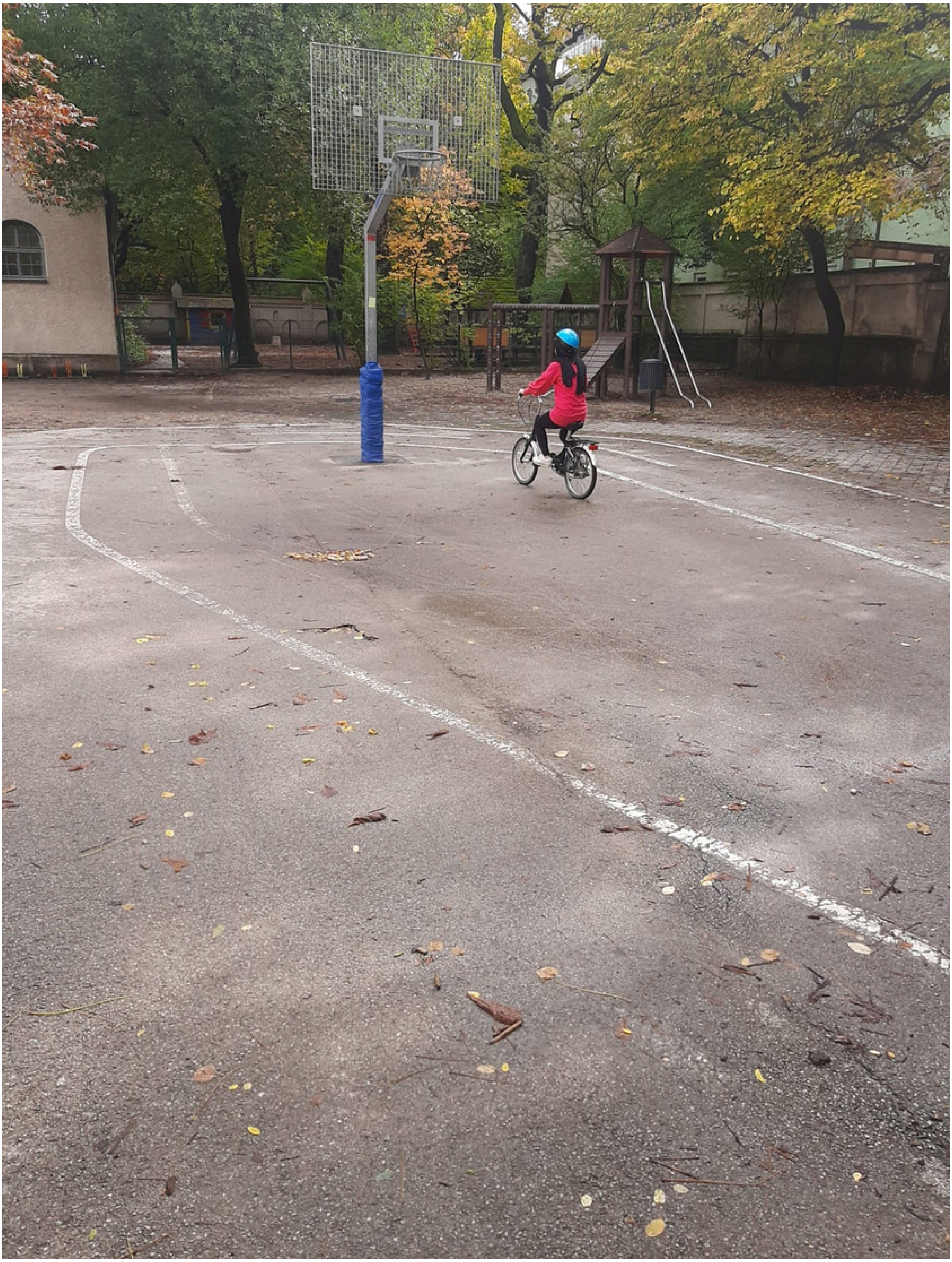

"It was so good, really. I was proud that I could do it."

Photograph 30

Title: **Balance from Thirty High**

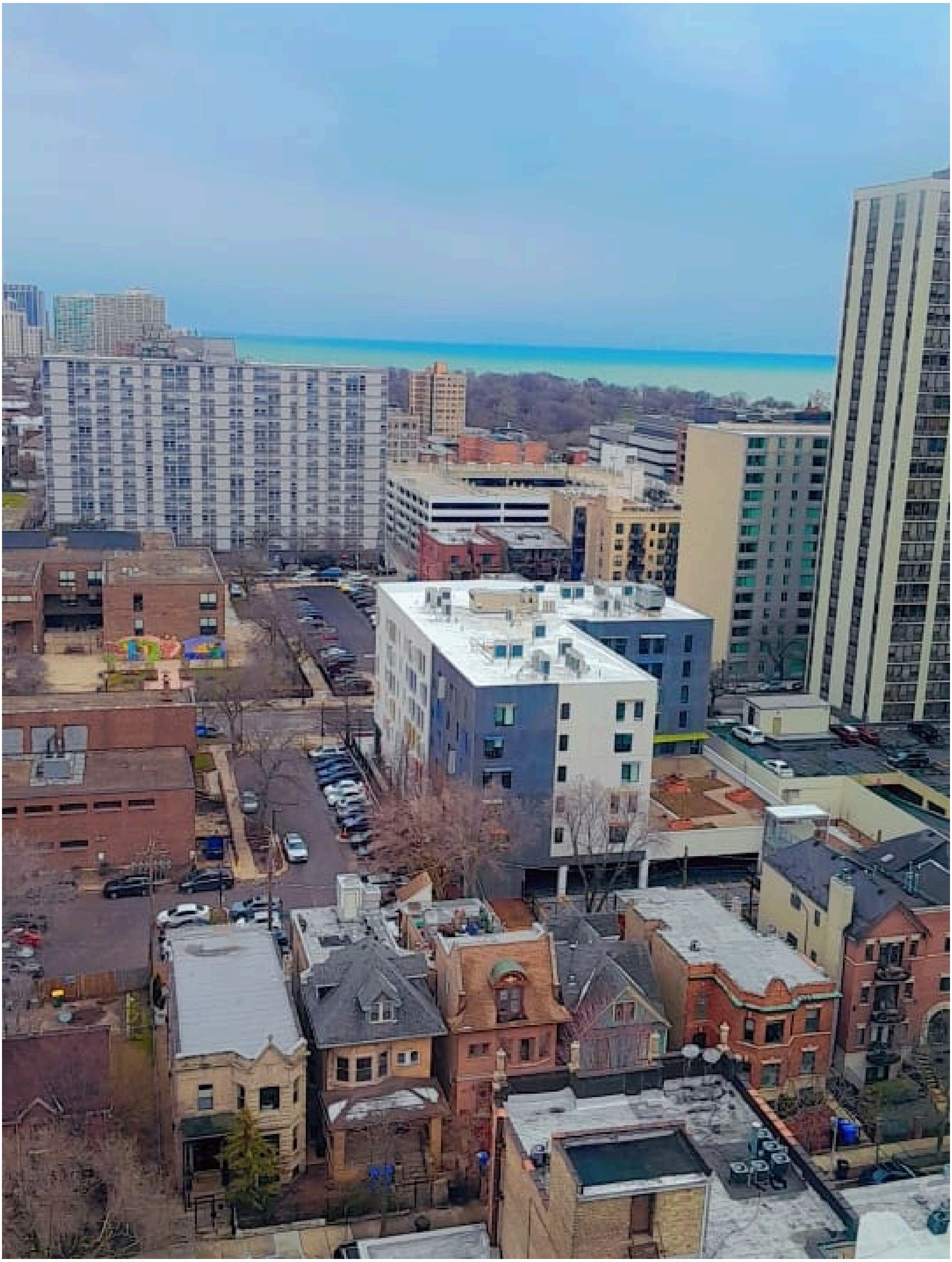

"Living by a lake and it's very cold. Today it is -2 degrees. […] This is the first time in my life that I have a irregular period because of the weather. Like I've been through a lot of things but for my period always there is no problem. But because of this weather I think it's because it's too much cold now I had the irregular period."

Photograph 31

Title: **Family Reflections**

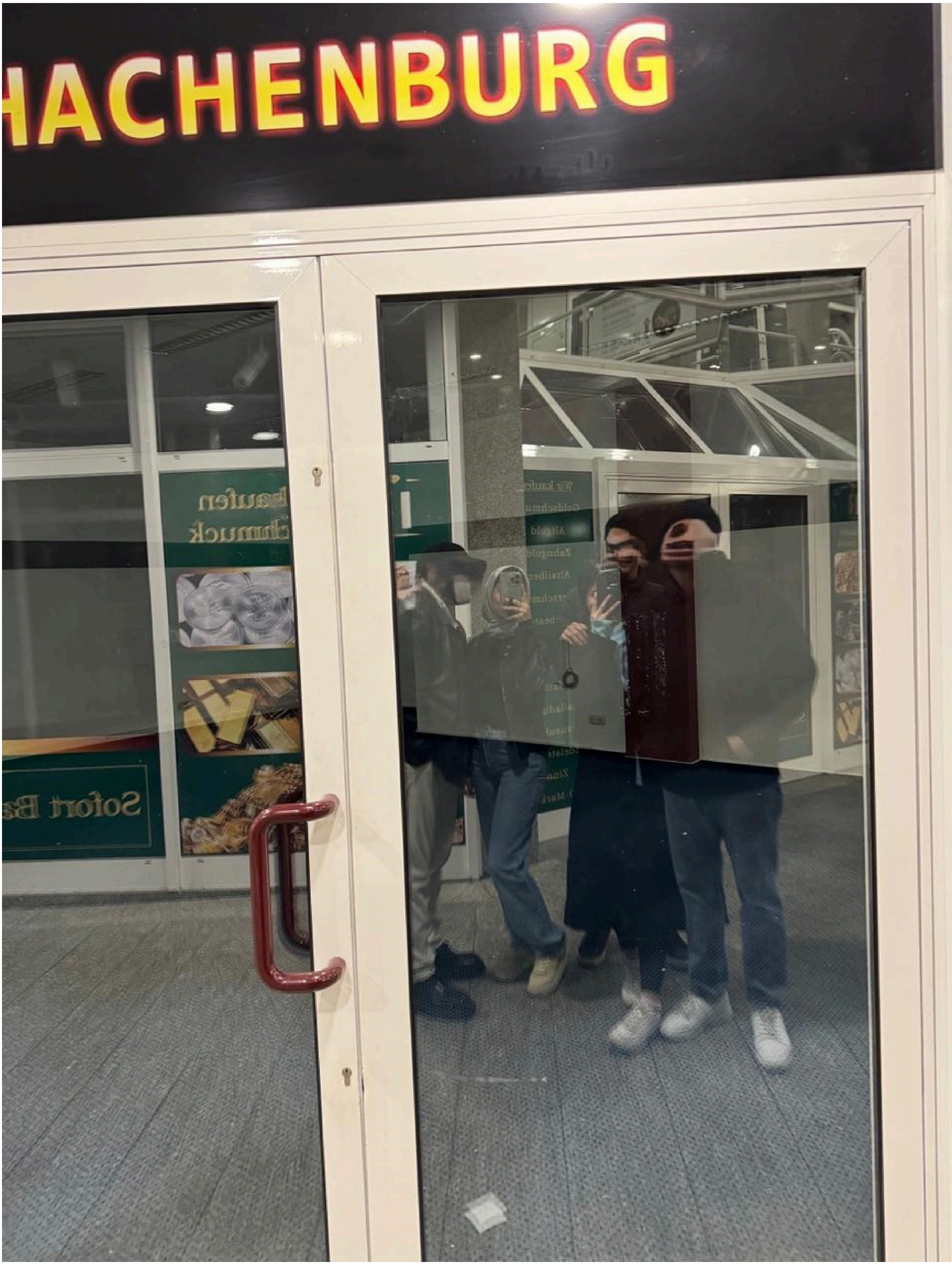

"This picture was yesterday with all my brothers and my fiancé, all of us together. It was my best moment for this week. I was very happy yesterday."

Photograph 32

Title: **The Future**

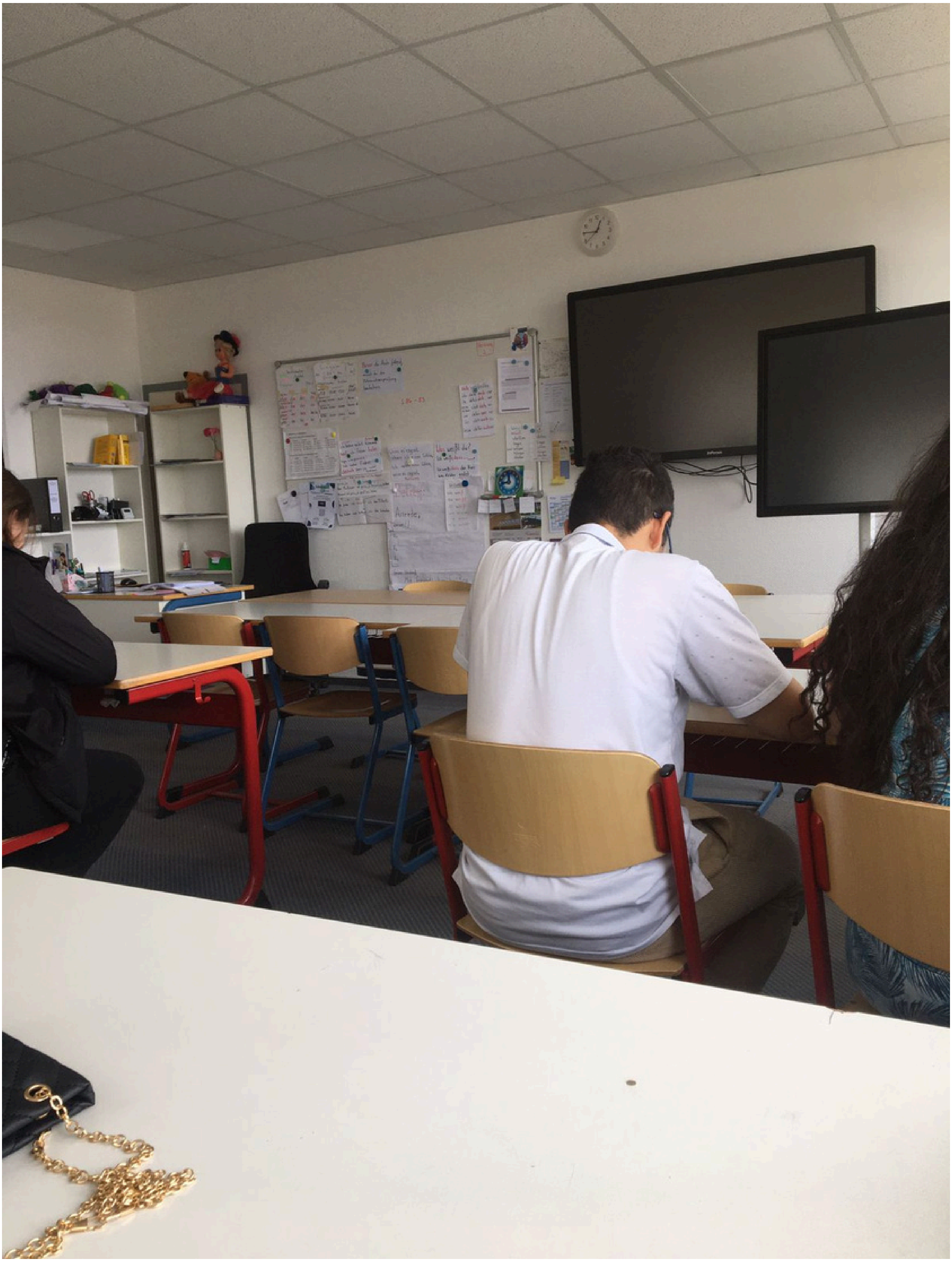

"Yesterday I had a language test, after waiting seven months, and a level was determined, and I will start in July. I was frustrated in the previous months, but now when the study date was set, it had a positive impact on me."

<u>Photograph 33</u>

Title: **Challenging Crossroads**

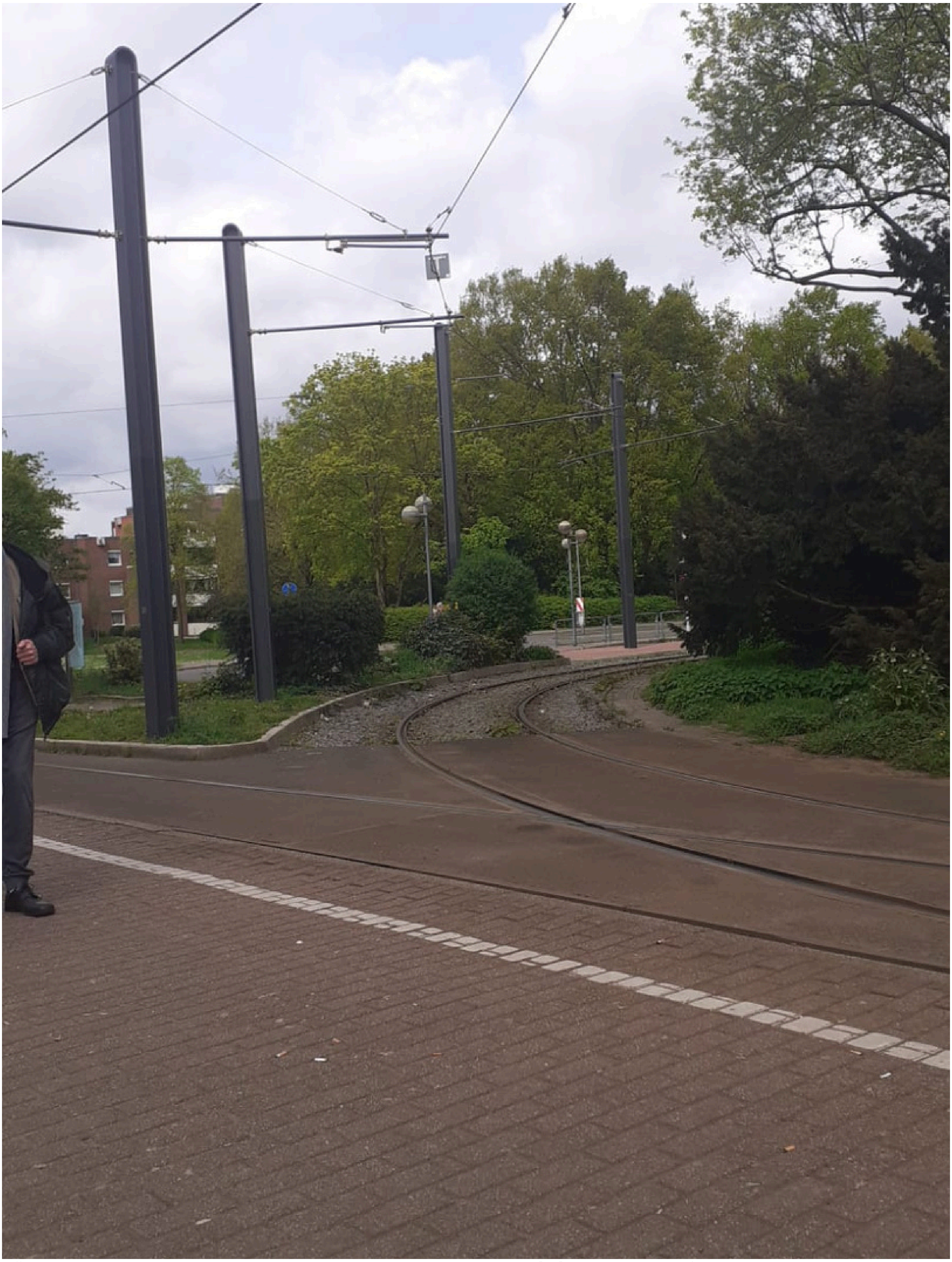

"Everything that happened to me was while I had my period […] I was looking for a job and in the camp and Alhamdulillah (all praise to God) I know a little bit of English so that supports me. You know mostly the people that don't speak English had a paper it's up to you to translate by yourself. If you want to, find job if you want to go anywhere, […] there is no place that guides you. Everything is by yourself. You have to do everything by yourself."

Photograph 34

Title: **Unity in Solitude**

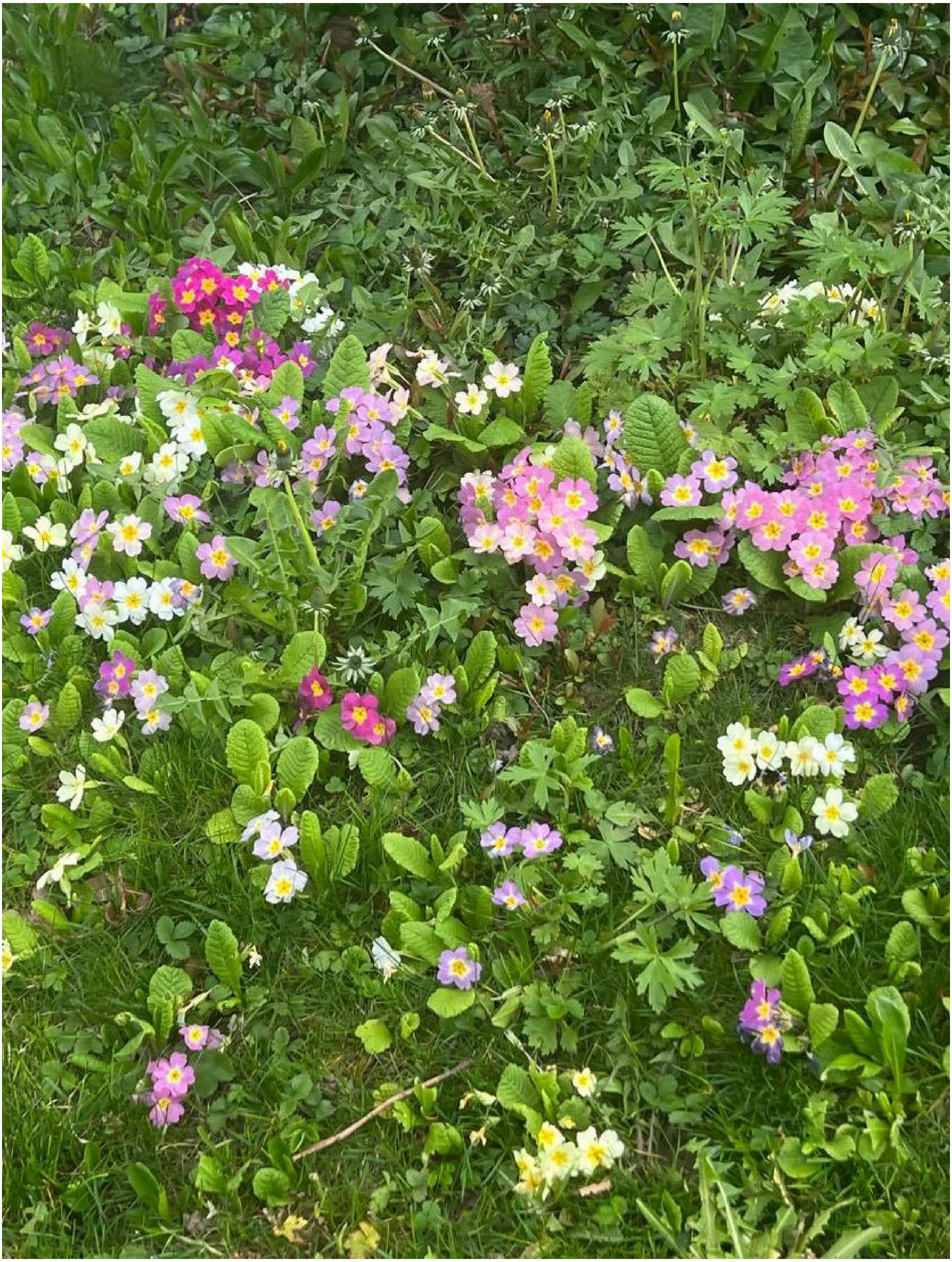

"I was thinking of our research group. When we were together, when we worked together. We have our own capacity, our own beauty. But when we made it work together, it became extraordinary for me. Because each one flower has its own beauty. But if there is no unity, I don't think it would be shining. Unity is strength."

Photograph 35

Title: **Space to Think**

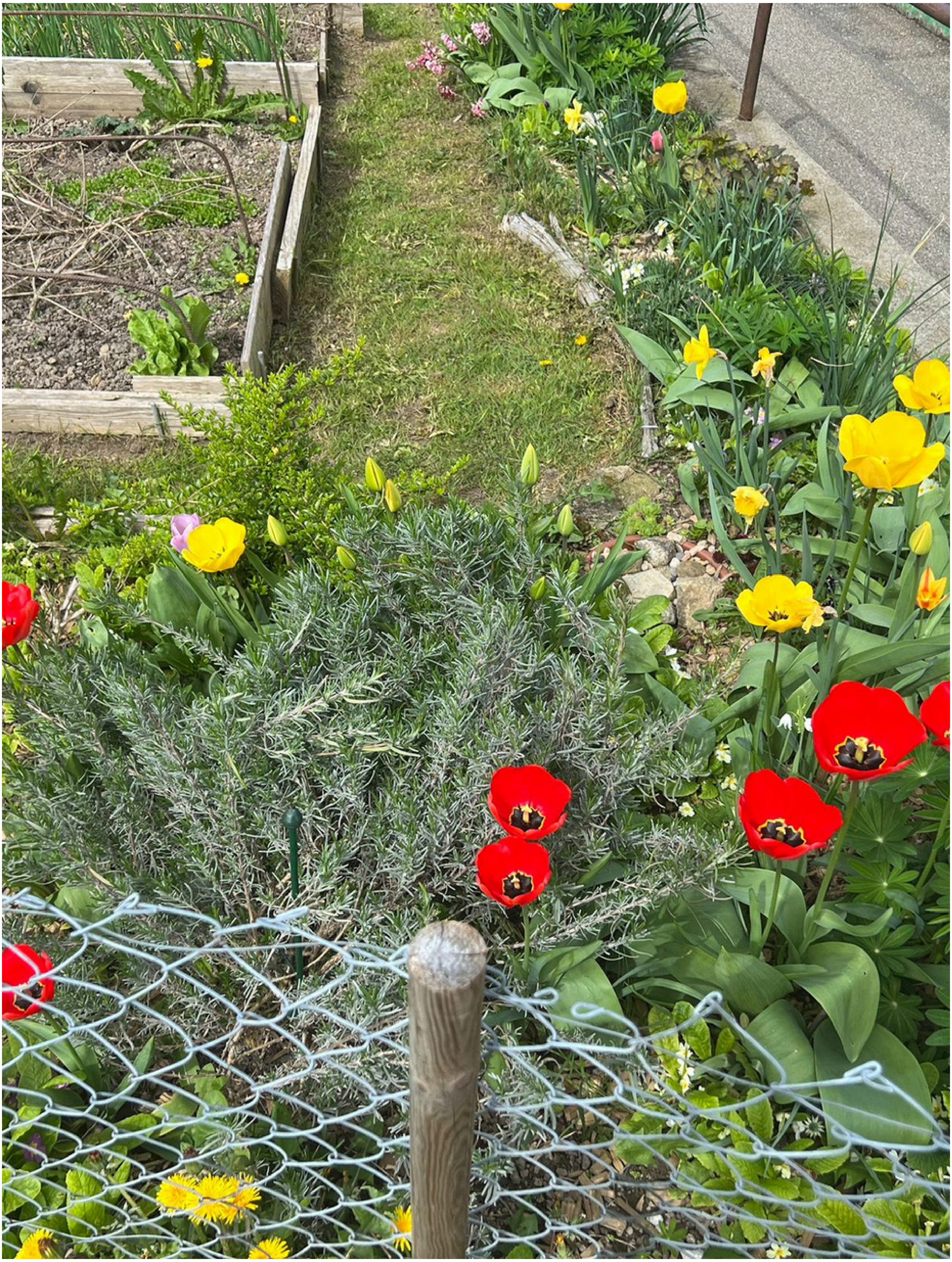

"When I see this picture it gives me good feeling. The first one with the grass and green things – like green things for me […] I found it something place like talk with yourself, having some time really to think and like to get some fresh air. […] if you want you can shout also. Like I'm stressed or something I'm going to go there and shout and then I think a time."

<u>Photograph 36</u>

Title: **Struggling to Survive**

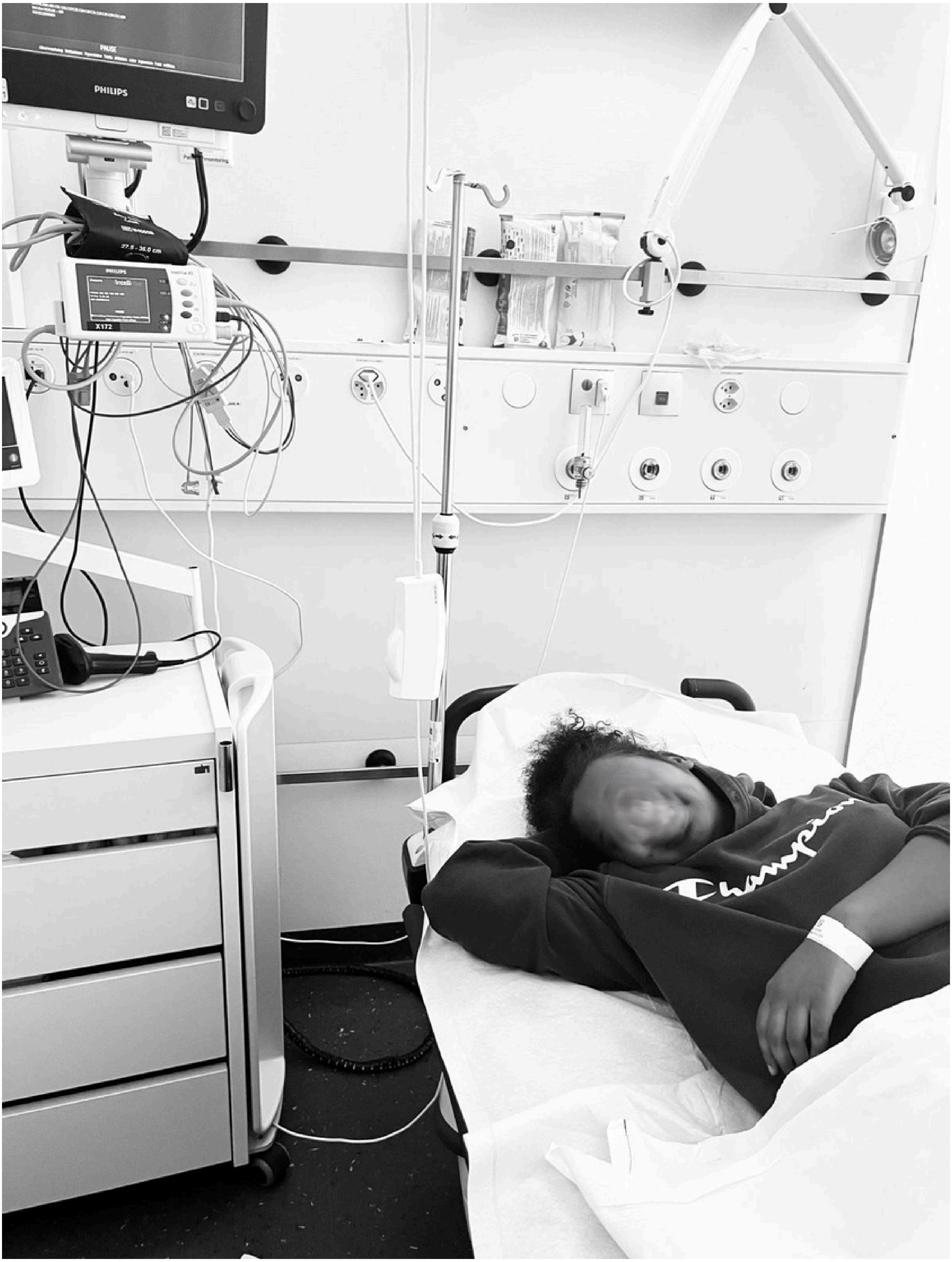

"You get sick when you feel safe."
