# Supplementary material for: Impact of Displacement on Refugee Women's Sexual and Reproductive Health: A Participatory Study Using Photovoice
Source: BJOG. 2025 Aug 19;132(13):2204–16. doi: 10.1111/1471-0528.18328 (PMC12592786; doi:10.1111/1471-0528.18328)
Supplement: Supplementary file 2 — Table S1: Qualitative analysis. [file BJO-132-2204-s001.docx]

| **Overarching theme** | **Code** | **SRH domain** | **Symbolism** | **Exemplary citation** | **Photographs** |
| --- | --- | --- | --- | --- | --- |
| **Bodily autonomy** | Exacerbation though displacement | Child marriage  Mental health |  | *“Like even some girls maybe she's fourteen or fifteen years old and the family they make like economic marriage… I have experienced some ladies when they are moving out of their countries, they get married because to get asylum. They don’t want to, they don’t like him, they don’t love, they just get married because of to get asylum and this makes like a different problem like even…mental health problems.”* | 2 |
|  |  | GBV  Mental health | Ocean | *“They told me that's how they scared now from the police, even when they see the police inside the camp because they have really bad memories from the Greek police. They told me how they have been checked by the male police guard even they checked their very - how can I tell - your personal areas and private parts of their body. So, because of that I sent you this picture and how this kind of memories caused some kind of mental problems for the women.”* | 11, 20 |
|  | Reaching one’s potential | FGM/C  Mental health | Blossoming flowers | *“I want to tell the parents not to do that. Like those flowers: if we keep them, our flowers could be beautiful - they can have a nice fresh air, everything.”* | 3 |
|  |  |  |  | *“Then when the flowers blossom yeah. If a girl doesn't experience FGM she could have pleasure, she could be more productive even when she give birth like she could be just… those leaves are the production like she can product even mentally.”* |  |
|  | Hope for treatment in resettlement country | FGM/C  Mental health |  | *“As I said before there's a lot of struggling with the FGM. You know some people they complaining that they lost all of their clitoris or every part of their vagina so that they don't have any reconstruction or any surgery according to the FGM but now here there is a surgery like free and yeah and the government support them if they need like to make a reconstruction.”* | 2 |
|  | Hope for protection of bodily autonomy in resettlement country | FGM/C | Flowers  Colour red | *“Last time there was a session about FGM in the centre and those flowers came to my mind to draw. In the sessions they were telling like in Switzerland it's illegal to have this FGM to cut for the ladies.”* | 2, 3 |
| **Instability** | Uncertainty about asylum | Mental health |  | *“You can see all the time like especially in the morning I can see a lot of people feeling sad and feeling fear when they have appointment with their lawyer to tell them the answer. Yeah, every morning there are a lot of people with sad faces.”* | 8, 9, 10, 23 |
|  |  |  |  | *“Yes, to the instability, so because this picture is more about like waiting for the interview or for the answer and then it's instability because you don't know what's going on.”* |  |
|  | Safety | Mental health  GBV | Ocean  Earthquakes  Mountains | *“Yeah, it feels more safe here. I think that there could be smaller oceans but not like in Greece or Turkey or Italy. It's good, I connected with myself with those flowers and sometimes with the mountains. Like even if you got here, you can see a lot of beautiful things like from the nature… Do you know in Athens there was earthquake one day? The whole Greece is sinking to the ocean. It doesn’t feel good for me. I was really eager to get out from that country. A lot of things happened there.”* | 7, 20 |
|  |  |  |  | *“In my mind it's like this: the sunsets and the sky is beautiful but in the contrast the sea can die the people. Even though the sea can make you feel calm and can give the people peace, but on the other hand it can be very dangerous and be like a murderer”* |  |
|  | Financial burdens | Mental health  Maternal health |  | *“She’s going to give birth and she had nothing and like my uncle is also so busy with this to take her to this hospital, and to this hospital, and to this hospital. Like to get insurance. […] But like you know? For her she's an asylum seeker she's not good mentally, she's not good physically, she's so tired still now and she had a lot of things like she's not talking. I don't know why she don't talk.”* | 5, 16, 17 |
|  |  |  |  | *“Having a paper or not this is something else. Whether I have it now or after one year, or two years, I’m not going anywhere else. I'm here so I need now having a money and paying all of my expenses and then having some stable life having new home and living like the others.”* |  |
|  | Waiting to find home | Mental health |  | *“Even though you’re in a house it still feels like a tent”* | 14, 23, 32 |
|  |  |  |  | *“A lot of people get crazy there. I know a lot of people they have a lot of beautiful dreams like they want to do something. But when they stay more than two or three years, lack of future... just stay and eating that food. Even that food is not food. There are no homes just plastic tents.”* |  |
|  | Importance of being able to work | Mental health |  | *“I can say it’s just very stressful if you can’t find a job. Like any environment you don’t have any idea what you going to do. Some people they don’t even know how to use the location, some people they don’t know how to use the transportation. It’s really stressful.”* | 4, 5, 33 |
|  |  |  |  | *“Like after the war broke out there was not any place for me in [home country] that’s why. I fight with the government they tried to kill me and I had to leave that’s why. […] To be honest I think I love being journalist, […] it's not easy to accept it everything is like it's not easy. Now like I already accept it, but first it was like that place it doesn't feel comfort for me. Like going from the studio to the kitchen it doesn't give sense for me.”* |  |
| **Living conditions**  *(note: was rephrased from initial ‘difficulties of camp’ that the participants identified as a theme)* | Lack of privacy | Mental health | Nature  Rain  Mountains | *“If you want to be sad, even if you want to laugh, there is no place here. Like even if I want to be happy I cannot be happy to be honest. Even if I want to laugh, maybe someone could cry next to me, so it doesn’t feel good. So if you want to, you have to control your emotions inside the house.”* | 7, 8, 23, 34 |
|  |  |  |  | *“When I see this picture it gives me good feeling. The first one with the grass and green things – like green things for me […] I found it something place like talk with yourself, having some time really to think and like to get some fresh air. […] if you want you can shout also. Like I’m stressed or something I’m going to go there and shout and then think for a while.”* |  |
|  | Shared toilets | Gyneacological health |  | *“You know I was knowing the time of cleaning, like at the morning, at the known afternoon I always go in like that type of cleaning you know? When they clean that time is really clean, so I was using that time mostly.”* | 2, 6 |
|  | Lack of freedom | Mental health | Fences | *“But for me it’s really very hard to me. OK if they allow me to just cook what I want! […] nobody can cook, you cannot even use hair dryer you cannot use anything you cannot even buy any machine […] if you want tea or coffee no. Just sleep and your clothes. Like the kettle that you use to make a coffee or a tea, just no. Just your clothes and your bed only. I don’t know it’s like a jail. I say this life is a jail to me for two or three years but alhamdulillah (all praise to God)”* | 22, 33, 35 |
|  |  |  |  | *“The fence always reminds me prison. I think that when there is a fence, I remind Moria. Moria and all camps.”* |  |
|  | Diversity of people | Mental health | Flowers | *“Yeah, but when I see this heart and let’s say in this camp there is a lot of ethnicities, like a lot of people from the whole world. We are different like those flowers but at least even when we disagree each other we can be kind to each other, right? Yeah, that symbolism comes to my mind. We can be kind to everybody, we can give flowers. You cannot lose anything by being kind.”* | 18, 34 |
|  | Weather changes | Menstrual health |  | *“Living by a lake and it’s very cold. Today it is -2 degrees. […] This is the first time in my life that I have a irregular period because of the weather. Like I’ve been through a lot of things but for my period always there is no problem. But because of this weather I think it’s because it’s too much cold now I had the irregular period.”* | 30 |
| **Social support** | Navigating medical healthcare |  |  | *“Oh yeah that picture I take it myself. Like my phone was with me it was the second day or third day? And then my friend – she’s so kind – she couldn’t sleep. I don’t know how say, she always keep an eye for me. She don’t even want me to sleep. Like if you sleep you’re going to die. And then at one moment when we were talking and she fell asleep and I take that picture.”* | 19, 21 |
|  | Navigating new careers | Menstrual health |  | *“Everything that happened to me was while I had my period […] I was looking for a job and in the camp and Alhamdulillah (all praise to God) I know a little bit of English so that supports me. You know mostly the people that don’t speak English had a paper it’s up to you to translate by yourself. If you want to, find job if you want to go anywhere, […] there is no place that guides you. Everything is by yourself. You have to do everything by yourself.”* | 33 |
|  | Nostalgia and transience | Mental health | Sunsets  Flowers  Fences | *“Most of the time when I see the sunset time I miss… I miss my family more than other times. […] It’s so beautiful but then when I see it, it makes me more sad than happiness”* | 4, 20,22, 35 |
| **Celebrations** | New home |  |  | *“Facilitator: So, what is the message behind all of these photos?*  *Participant 5: It’s like celebration or happiness.*  *Participant 6: It’s like growing in your new home. Having a new home after being on the move for so many years maybe.”* | 25, 26, 27, 31 |
|  | New skills |  |  | *“(about a picture of Participant 6 on a bike) It was so good, really. I was proud that I could do it.”* | 29 |
|  | Relationships |  |  | *“This picture was yesterday with all my brothers and my fiancé, all of us together. It was my best moment for this week. I was very happy yesterday.”* | 31 |
|  | Achievements |  |  | *“I took it last night because it’s after our C1 test and we were too busy busy busy and tired, and I was relaxed completely, and I took this picture, and I was happy.”* | 25, 27, 32 |
| **Healthcare access**  *(note: was rephrased from initial ‘barriers’ and ‘difficulties of moving to a new place’ that the participants identified as themes)* | Less priority given to health | Gynaecological health |  | *“Yes, I have to give the first priority for my health. But in this moment I can't because the first thing is I have to give the priority for my work.”* | 5, 16, 17 |
|  | Feeling safe enough to fall ill |  |  | *“Last Sunday Monday Tuesday I was in hospital. I was sick. Now I’m ok. I don’t know it’s my first time to be like this in my life. Suddenly I couldn’t breathe. It was in the middle of the night.”* | 19, 36 |
|  | Health education | FGM/C |  | *“Let's say mostly the people they can’t write or they can’t read so even if they maybe couldn't get a chance of high school or just academic process so […] if they just have a look like what you can get so I think this makes like a healthy promotion or just like health education.”* | 2, 3 |
|  | Language barriers | Maternal health  Family planning |  | *“I help her I’m not complaining, but you know it’s really their [asylum centre] responsibility you know. This is their job is not my job. These people I’m not hosting them. If I hosted people yes, I have to do everything you know.”* | 1, 25, 32 |
|  | Physical barriers | Maternal health |  | *“And that’s why I chose this picture. The reason that there is also stairs in this picture it’s difficult for the women that they had delivery to go up from the stairs and reach the toilets and bathrooms.”* | 6 |
|  | Financial barriers | SRH generally  Gyneacological health |  | *“Also when you receive a negative answer then you don’t have any insurance anymore and then for some of the problems if you need to visit a doctor in hospital then you can’t go there because you don’t have insurance. [This is] one of the other barriers that women cannot access the good sexual and productive healthcare.”* | 9, 16, 17 |
|  |  |  |  | *“I was in the hospital, and I have no insurance and here if you want to go to the hospital you have to get an insurance.”* |  |
|  | Long waiting times | Gynaecological health |  | *“Also the long lines. If it was today for example, if they want to visit a doctor about problem that they have and also a women’s doctor for example, again gynecologist or something like this, they have to wait in the long lines for so many others and also maybe so many weeks and days until they can have an appointment with this kind of doctors.”* | 13 |
|  | Navigating new systems |  |  | *“So, most of the pictures at the hospital have been taken by Participant 2 or Participant 1 and they just arrived in their new place. This can be how difficult is…this way of moving to arrive to a new place. Especially the times at the beginning.”* | 33 |
|  | Undignified treatment |  |  | *“He didn’t scan anything [while] I was seeing him, and he didn’t explain anything to me and he didn’t treat me well even. I really felt frustrated and really disrespected you know? But just thinking if you’re a refugee and you don’t know anything. You don’t even know how to type on Gmail or anything.”* | 15, 33 |
|  |  |  |  | *“I think they think that refugees not study… they don’t know anything. Because some people, where they sitting now, I was working before! And I don’t know they treat me bad. I don’t know.”* |  |
|  | Symptoms not taken seriously | Maternal health |  | *“And my friend […] calls the workers. And they check my thermometer my BP and they said: you are ok, there is no emergency. And I said yes, I don't have any fever but something immediately happened to me. I was being in my home, I was doing my normal work, I was ok, I didn't have this pain. It’s the first time my eyes get dark I feel like this dizzy frightening feeling. So, how you can explain all of this stuff?”* | 33 |
|  |  |  |  | *“I saw when the ambulance came like a pregnant woman, they give birth…like I saw it came out like she gave birth in the ambulance.”* |  |
|  | Attention to mental health | Maternal health  Family planning  Mental health |  | *“And then doctor said she's a pregnant and the doctor’s listening her heart pumping with the baby and she's crying a lot. […] The only thing I’m really confused, there is no psychologist or some stuff. Because the lady was pregnant she gets stressed.”* | 1 |
|  | Cultural sensitivity | Maternal health  Family planning |  | *“And one lady [the social worker] she said: if she doesn’t want to keep the baby, I can take the baby. I can keep her and I can take the baby. I don’t know because this is not our…you know, I see that nobody just respects you and nobody treated your culture, and nobody treat your tradition. Like you know, if I'm not respect your tradition, if not more respecting culture, it's like I don't care you! It means I'm not respect you.”* | 1, 28 |
| **Resilience** | Patience |  | Nature  Different colours | *“It’s like, I was thinking, the timing is just like the nature. Sometimes even the landscape by itself. When the winter comes and we lose the beauty of the landscape and now it’s all about the timing. If we really want something maybe for the time being we can lose but it will come at the right time.”* | 7, 33 |
|  |  |  |  | *“You know Allah says this life is hard for the moment. So, it's hard for us, and I accept it and I'm saying Alhamdulillah. That’s it. Yeah, its normal. Just for two years and a few years later. Its temporary.”* |  |
|  | Perseverance | FGM/C  Menstrual health | Flowers  Fences | *“Like these flowers. In the world there is no space for flowers, but it’s just rising.”* | 24, 14 |
|  | Feeling safe enough to fall ill | General SRH |  | *“Me and Participant 6 had this experience as well. You get sick when you feel safe. For example, Participant 2 she just arrived in [country], and she wanted to stay there and she felt herself safe so then she got sick. And it can show how difficult it was for example these two or three years that she was unmoved but because she had to be strong and don't get sick until she can be in a safe place. You know it happens to most of the people when they arrive after some years of being on move. In the way like it happened for me as well, I I've been to the hospital, I went to the hospital. It happens to most of the people.”* | 16, 36 |
|  | Multiple burdens | Menstrual health  FGM/C |  | *“You know for me I have FGM and when I get my period it’s super pain! Sometimes I start crying, crying until I get sleep. Its super painful for me. […] Even when I was traveling, I had my period. Can you imagine? Even that day when my dad died, I had my period. Sometimes I hate my period, I don’t know if it’s my bad luck. [That day I was looking for a job too.] They just written down your name and nobody talk to me, I just standing there for around one hour and nobody talks to me. Everybody works inside the company and I’m outside. I sometimes see the staff walking, and nobody talk to you. There is no office, no welcome office, nothing.”* | 33 |
| **Finding purpose** | Recognition | Family planning  Maternal health  Mental health |  | *“She said are you kidding? I said: No, there's a baby! I see the placenta! I see everything because in [my home country] I was working with the ultrasound. […] I always like to learn something with the health science, and I know a little bit with the ultrasound, and I see the placenta I said I’m not kidding there is a baby! And the doctor said: yes there's a baby.”* | 1, 4, 5, 28, 33 |
|  | Feeling valuable |  |  | *“It really feels good when the people say you make something and say it's so sweet even when they come when they smell it like yeah. It feels good.”* |  |
|  | Work |  |  | *“Yeah, it’s hard work… it’s not my field but you know just waiting and not doing anything. It’s better to just go and work.”* |  |
